# Fecal Microbiota Transplantation in Reducing Uremic Toxins Accumulation in Kidney Disease: Current Understanding and Future Perspectives

**DOI:** 10.3390/toxins15020115

**Published:** 2023-01-31

**Authors:** Gianvito Caggiano, Alessandra Stasi, Rossana Franzin, Marco Fiorentino, Maria Teresa Cimmarusti, Annamaria Deleonardis, Rita Palieri, Paola Pontrelli, Loreto Gesualdo

**Affiliations:** Nephrology, Dialysis and Transplantation Unit, Department of Precision and Regenerative Medicine and Ionian Area (DiMePRe-J), University of Bari “Aldo Moro”, Piazza G. Cesare 11, 70124 Bari, Italy

**Keywords:** fecal microbiota transplantation, PBUTs, chronic kidney disease, acute kidney injury, kidney transplantation, uremic toxins, oral FMT

## Abstract

During the past decades, the gut microbiome emerged as a key player in kidney disease. Dysbiosis-related uremic toxins together with pro-inflammatory mediators are the main factors in a deteriorating kidney function. The toxicity of uremic compounds has been well-documented in a plethora of pathophysiological mechanisms in kidney disease, such as cardiovascular injury (CVI), metabolic dysfunction, and inflammation. Accumulating data on the detrimental effect of uremic solutes in kidney disease supported the development of many strategies to restore eubiosis. Fecal microbiota transplantation (FMT) spread as an encouraging treatment for different dysbiosis-associated disorders. In this scenario, flourishing studies indicate that fecal transplantation could represent a novel treatment to reduce the uremic toxins accumulation. Here, we present the state-of-the-art concerning the application of FMT on kidney disease to restore eubiosis and reverse the retention of uremic toxins.

## 1. The Gut Microbiome in Health and Kidney Disease

The healthy gut microbial ecosystem consists of trillions of microorganisms that play a pivotal function in maintaining homeostasis by influencing metabolic, oxidative, and cognitive status and immune defense against pathogen infections. Each individual displays a unique microbial profile in early life depending on their gestational date of birth, type of delivery, milk feeding methods, sex, and gender. In adulthood, this healthy native microbiota remains relatively stable, despite several factors including body mass index (BMI), exercise, dietary habits, pharmacological therapies (e.g., antibiotics), and aging that can alter its composition [1]. According to large-scale studies, higher microbial diversity and richness in phyla, genera, and families is associated with healthier and advantageous intestinal status [2,3]. More specifically, the abundance of some enterotypes as *Bifidobacterium Bifidum*, *Lactobacillus acidophilus*, or *Streptococcus thermophilus* has been widely described to be beneficial for an effective immune response [4,5]. Furthermore, bacteria belonging to *Clostridiaceae*, *Bifidobacteriaceae*, and *Bacteroidaceae* families have been found in microbial communities of centenarians, suggesting that this phenomenon could reduce the age-related immune system dysfunction [6].

In this scenario, dysbiosis represents both a structural and a functional alteration of the microbiome that is closely associated with a specific disease. Importantly, the comparative metagenomic analysis shows the differences in gut microbiomes profiles between pathological and non-pathological conditions. For example, chronic diseases (i.e., rheumatoid arthritis, inflammatory bowel disease (IBD), diabetes, etc.) are characterized by lower microbial diversity and richness, higher levels of harmful bacteria, and an abnormal *Firmicutes*/*Bacteroidetes* ratio [7,8,9]. On the other hand, these studies also suggest that differences between a microbiome profile occurs between diseases.

Over the last decade, the mutual crosstalk between the gut microbiome and human disease enticed growing consideration in numerous intestinal and extra-intestinal diseases, such as chronic inflammatory diseases, metabolic dysfunction, neurological disorder, and cardiovascular disease [10]. In the context of renal disease, it still needs to be clarified whether intestinal dysbiosis represents a cause or a consequence, since a noxious cycle was recognized between uremia and gut microbiome [11]. A large plethora of data established that the alteration in the microbiome arrangement represents a consequence of kidney injury and strongly drives its exacerbation due to the accumulation of manifold bacterial-derived toxins [11,12].

On the whole, the uremic intestinal community is typically marked by the high proportion of *Actinobacteria, Bacteroides*, and *Firmicutes,* which is rarely described in healthy conditions [13]. According to many metagenomic studies, CKD patients show alterations in the expression of 16S rRNA associated with the *Enterobacteriaceae* family (e.g., *Enterobacter*, *Klebsiella,* and *Escherichia* genera), indicating that the Gram-negative *Proteobacteria* represent a fundamental constituent of uremic flora [13,14]. The abundance of urease, which is uricase accompanied by tryptophanase-tyrosine phenol-lyase positive bacteria (e.g., *Actinomycetia*, *Methylococcaceae*, *Micrococcineae*, *Pseudomonadales*, *Alteromonadales*, *Micrococcales*, *Halomonadaceae*, and *Pseudomonadaceae),* was recognized as the hallmark of uremic dysbiosis, due to its proteolytic activity in producing uremic toxins [15,16]. Additionally, elevated growth of *Bacteroidaceae* and *Clostridiaceae* has been associated with systemic inflammation [17]. On the other hand, the strong reduction in the relative proportion of both *Lactobacilli* and *Actinobacteria* phylum together with the lower proliferation of *Prevotellaceae* and *Bacteroidacee* families reflect a decline in short chain fatty acids (SCFAs) production [17,18]. Of note, it has also been shown that differences in gut microbiome profiles occur not only between CKD stages but also between kidney diseases characterized by different phenotypes patterns. For instance, it was shown that hemodialysis (HD) patients exhibit a disproportion in *Gammaproteobacteria* and *Firmicutes* when compared with pre-dialysis patients [13,19]. Furthermore, IgA nephropathy (IgAN) seems to be characterized by the high amount of many microbial groups, including *Streptococcus* and *Paraprevotella* [20,21]. Interestingly, several studies indicated that the enrichment of *Escherichia-Shigella* is increased in diabetes-associated kidney damage [22]. On the other hand, the overgrowth of *Anaerosporobacter* and *Blautia* was associated with metabolic dysfunctions in diabetic nephropathy (DN) [23,24]. Interestingly, recent evidence has indicated that intestinal dysbiosis also occurs in acute kidney injury (AKI) [25]. For instance, Andrianova et al. demonstrated by an in vivo study that alterations in the microbiome composition occurred following renal ischemia/reperfusion injury (IRI), and several bacteria including *Rothia* and *Staphylococcus* were linked to the high degree of injury [26]. In line with this research, Yang and their co-workers showed that the AKI-related microflora contributed to the exacerbation of renal damage, inflammation, and intestinal permeability when transplanted in germ-free animals [27]. Finally, more recent data highlighted the involvement of dysbiosis and uremic toxins after solid organ transplantation, including kidney transplantation. In detail, the 16S analysis of renal transplanted patients detected a profound disruption of microbial diversity associated with the enrichment of uremic toxins producing *Proteobacteria* and *Enterobacteriaceae* [28,29]. Based on this evidence, strategies aimed to restore eubiosis and the levels of microbiome-related metabolites could represent a promising therapy for kidney disease. However, an alteration in microbiome composition is not necessarily negative; microbiome composition can be modified and accommodated by interventions such as the Mediterranean diet, supplements (probiotics, prebiotics, and Ω-3 Fatty Acids) or exercise that influences the inflammatory state, which can decelerate CKD progression [30,31,32].

The growing knowledge of the detrimental effect of dysbiosis in kidney disease has supported the development of several targeted strategies to restore the level of uremic toxins. In the first instance, the therapeutic interventions based on biotic supplements aimed to prevent the generation of PBUTs by rebalancing the gastrointestinal flora equilibrium. However, a great number of conflating elements, such as the period of administration, bacterial amount, and strain selection impede the result interpretation and the method standardization. In this scenario, the manipulation of microbiota by FMT could represent a novel treatment to reduce the uremic toxicity in patients with CKD. Therefore, this extensive review highlights the current knowledge of the role of fecal transplantation in the context of kidney disease, providing novel insight into the FMT-based strategy to correct the levels of uremic toxins.

## 2. The Gut–Kidney Axis

**Figure 1:**
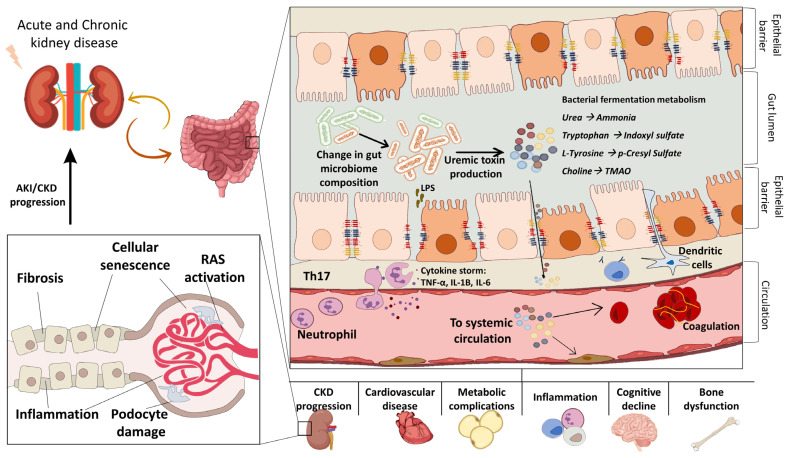
The detrimental effect of microbiota-derived uremic toxins accumulation related to kidney disease.

When the glomerular filtration rate decreases, a considerable amount of nitrogenous toxic catabolites (i.e., urea and urates) accumulate into the blood of CKD patients [33]. Against this background, the removal of these toxins is supported by the intestine, resulting in their retention in the gut lumen. As a result, the uremic milieu promotes a sustained dysbiosis characterized by the disequilibrium of proteolytic bacteria to the detriment of the saccharolytic communities. Consequently, the strong urease activity along with intensive proteolytic fermentation enhances the conversion of urea and amino acids (e.g., tyrosine, tryptophan) in toxic compounds, named uremic toxins [11]. Several studies demonstrated that the retention of such compounds strongly affects the integrity of gut mucosa by triggering a leaky gut and local inflammation. In line with this evidence, a marked impairment of epithelial junctions including CLDN, OCLN, and Zonula occludens-1, was observed within the intestinal barrier of several animal models of CKD [34,35,36]. Additionally, the translocation of a huge number of toxins in the circulation together with the triggering of the immune response branch causes a systemic hyperinflammation and exerts multi-organ damage [37] (Figure 1).

### 2.1. Uremic Toxins in CKD

Uremic toxins figure as the chief contributors to the development of uremic complications during acute or chronic kidney impairment and they harm several physiological functions. According to their molecular weight and characteristics, they are usually categorized into small solutes (<0.5 kDa), middle-weight molecules (0.5–60 kDa), and protein-bound uremic toxins (PBUTs) [38]. Small solutes (creatinine, urea) are usually successfully removed by the conventional hemodialysis techniques. The second category includes peptides and proteins with middle-molecular weight molecules, such as b2 microglobulin and alfa1-macroglobulin. In patients with a normal kidney function, renal elimination accounts for 30–80% of total removal, while during renal injury, the removal of such compounds may be significantly altered. The final category of uremic toxins (PBUTs) includes relatively low molecular weight molecules that present specific ionic or hydrophobic characteristics through which they strongly bind to albumin in the blood [39,40]. In patients with a normal kidney function, they are usually eliminated by organic anion transporters (OATs) in the proximal tubules [41]. Although conventional hemodialysis is the main technique used for the reduction of uremic toxins, it has been demonstrated that it is most effective in eliminating small water-soluble compounds, while the removal of middle-weight compounds and PBUTs is very limited (reduction rate <30–35%), due to the strong protein-bond of such compounds and the usual pores’ cutoff of low-flux (LF) membranes that avoid albumin loss and the consequent hypoalbuminemia [39,42]. Moreover, the increase in the number of HD sessions and/or the treatment time may improve small and middle molecules removal, but not for PBU molecules. Only the unbound portion of PBUTs could be efficiently removed by HD, due to their low molecular weight [39,43,44]. The reduction rate of PBUTs by conventional HD is listed in Table 1. Interestingly, most of the gut-derived uremic toxins including indoxyl sulfate (IS), p-cresyl sulfate (PCS), p-cresyl glucuronide (PCG), indol-3-acetic acid (IAA), and hippuric acid (HA), belong to the PBUTs group. On the other hand, the bacterial metabolite Trimethylamine-N-Oxide (TMAO) is grouped as small water-soluble molecules. The metabolic pathways of the most relevant uremic toxins together with their characteristics are summarized in Table 1. Briefly, phenol-derived PCS and PCG originate from tyrosine metabolism, while IS and IAA derive from tryptophanase-positive bacteria. Notably, tryptophan metabolism was also implicated in the kynurenine pathway [45].

The toxicity of PBUTs has been well-documented in a plethora of pathophysiological mechanisms in kidney disease, such as cardiovascular injury (CVI), metabolic dysfunction, and inflammation [64,65].

In the past years, mounting studies have corroborated the role of PBUTs in cardiovascular dysfunction and capillary rarefaction. For instance, in vivo and in vitro experiments established that indoles and phenols exert a role in vascular leakage by promoting apoptosis and affecting the integrity of the adherent junctions in endothelial cells [66,67]. Additionally, more recent evidence indicated that increased oxidative stress represents the most relevant consequences of endothelial damage in patients displaying the highest level of uremic toxins [68]. At the cardiac level, gut-related toxins were determined to trigger reactive oxygen species (ROS) origination by upregulating the NADPH oxidases (NOX) activity [69]. This finding was closely associated with gap junction damage in cardiac muscle cells resulting in cardiomyocyte dysfunction [70]. According to several in vitro studies, PBUTs modulate the endothelial cells senescence by downregulating the expression of klotho leading to vascular hypertrophy via the endothelial/mesenchymal transition [71,72].

The effect of several uremic toxins on vascular dysfunction may also be due to their ability to modulate the downstream expression of many intracellular miRNAs in endothelial cells [73,74]. Altogether, this evidence highlights that uremic toxins may represent the missing player between endothelial damage and uremia-related CVI. On the other hand, PBUTs induced detrimental consequences on systemic inflammation. For instance, Ito et colleagues, evaluated that the over-expression of E-selectin was mediated by PCS via JNK/NF-kB pathways, which resulted in the enhancement of leukocyte rolling on endothelial cells and organ extravasation [75]. More recently, several groups have found a cause–effect link between uremic solutes and the expression of certain pro-inflammatory cytokines [76]. At the renal level, the accumulation of PBUTs was demonstrated to increase the ROS levels in renal tubular epithelial cells (TEC) and mesangial cells by upregulating the NOX activity [77,78,79,80]. Furthermore, it was shown that the alteration of several cellular pathways including AhRs, NF-kB, and p53, in response to the elevated level of PBUTs, was associated with interstitial fibrosis and renin-angiotensin system (RAS) activation [49,81,82]. Moreover, klotho downregulation represents the hallmark of AKI and CKD and is strongly correlated with the senescence of renal cells [83]. The growing data suggest that PBUTs are involved in klotho hypermethylation by affecting the expression of several methyltransferases, probably via an NF-kB-mediated mechanism [84].

Finally, many experimental data found a strong relationship between uremic dysbiosis and CKD-related insulin resistance (IR) [85]. IR is indeed recognized as an important clinical condition that affects metabolic complications, sarcopenia, and cardiovascular injury in the CKD population. Moreover, the alterations in insulin signaling in CKD turns into kidney lipodystrophy, which is characterized by adipocyte dysfunction [85]. It is worth noting that adipocytes stimulated with blood from CKD subjects fixed an IR phenotype characterized by an impaired glucose uptake [86]. Additionally, Stocker-Pinto et al. demonstrated that an ROS imbalance occurred when the adipocytes were stimulated with uremic solutes [87]. To elucidate this mechanism, Koppe et al. demonstrated that IR together with the redistribution of body fat was detected when healthy mice were treated with PCS [59]. Altogether, these data indicate that PBUTs seem to be the missing key in exacerbating the metabolic complications in uremia. Interestingly, some of the uremic mediators related to the decline of kidney function seem directly associated with cognitive decline. For instance, the elevated accumulation of several small water-soluble solutes including uric acid, guanidino compounds, and asymmetric dimethylarginine (ADMA), were found to be implicated in neurotoxicity [88,89,90]. Additionally, many studies indicated that higher levels of IS and PCS increased neuroinflammation, resulting in the cognitive impairment of patients with CKD [91,92]. Moreover, indole and cresol can cause BBB impairment via AhR activation, leading to inflammation and oxidative stress [92]. Homocysteine (Hcy), which is produced through the intestinal metabolic transmethylation of methionine to cysteine, has been linked to neuronal damage in many neurological diseases. Patients with CKD who show elevated concentrations of plasma Hcy are known to suffer from cognitive and motor impairment [93]. The proposed mechanism involves the overstimulation of the N-methyl-D-aspartate receptors (NMDAR) [94]. Neuroinflammation represents another detrimental factor in CKD-associated cognitive impairment, since pro-inflammatory mediators (IL-1β, IL-6, TNF, and TGF-β) and immune cells exacerbate the cognitive decline in CKD patients [95]. The disruption of BBB during inflammation allows the interaction of cytokines with the neurotrophic factor (BDNF) in the central nervous system (CNS). Recently, the kynurenine pathway was found to be strongly implicated in the context of brain disorders. Several neuroactive metabolites can be catabolized from tryptophan, including kynurenine, 3-hydroxykynurenine (3HKYN), picolinic acid, and quinolinic acid (QUIN) [96]. The condition of inflammation can drive the upregulation of the kynurenine pathway, which lowers the synthesis capacity of serotonin from tryptophan. Elevated levels of pro-oxidative 3-HKYN in the CNS lead to increased neuronal apoptosis, due to its involvement in reactive oxygen species (ROS) formation, via superoxide and H_2_O_2_ generation [97,98]. QUIN is produced by microglia and penetrated by macrophages [99]. Excessive synthesis of QUIN leads to ROS formation via the excitation of the NMDA receptor [100,101]. This increases lipid peroxidation, nitric oxide levels, protein decomposition, and cytoskeletal destabilization [101].

### 2.2. Uremic Toxins in AKI

Compared with CKD, interest in uremic toxins in AKI was in its infancy in 2009, and the existence of gut–kidney crosstalk in AKI was frequently debated. Today, it is well-established through a variety of experimental animal studies and clinical trials that acute renal damage negatively shapes the microbiota composition [25]. Importantly, the AKI–microbiota relationship is bidirectional: on the one hand, AKI can cause dysbiosis; on the other hand, the microbial shift influences the severity of the renal injury. To corroborate this theory, Jang et al. firstly demonstrated that after IRI induction, germ-free rodents displayed the worst creatinine and histological damage when compared to the controls [102]. Next, in the context of renal IRI, hypoxia itself can alter the ratio between aerobic and anaerobic populations [103]. More recently, Andrianova et al. demonstrated by an in vivo study that alterations in the microbiome composition occurred following IRI [26]. The connection between uremic toxins and an AKI occurrence has been strengthened by several studies [94]. Indoles and cresols are well-known toxins, and their circulating level has been connected with CVI and poor survival rates in end-stage renal disease [104,105]. Interestingly, the rising concentration of PBUTs in AKI is correlated with the severity, according to the RIFLE criteria [106]. In AKI rodents, the depletion of IS and PCS production alleviated the renal damage [107]. As already discussed, the gut microbiota composition is of pivotal importance, but not only for uremic toxins, as it can also influence immune responses. In addition, germ-free AKI rodents showed a higher activation of NK cells and CD-8+ cells compared with non-sterile controls. More specifically, the “sterile immunity” showed the over proliferation of the Th17 population. Th17 lymphocyte affects the kidney dysfunction by releasing the interleukin-17, which triggers a strong inflammatory response in the renal environment [108]. Moreover, the gut barrier could also be injured during AKI as the concentrations of uremic toxins increase. Additionally, circulating endotoxin levels stimulates a systemic and inflammatory effect. Against this scenario, one of the most recent interventional studies aiming to modulate AKI by uremic toxins/microbiome modulation was performed by Dong et al. [109]. The authors showed that antibiotic treatment was able to ameliorate the severity of acute renal damage.

In conclusion, while uremic toxins could represent the essential factors of AKI, there is only a limited amount of data available, as the majority of findings are derived from experimental animal models, which have severe limitations. Moreover, despite the advancements of dialysis technologies, the poor prognosis of AKI patients could be connected with microflora-related compounds, which exert multi-organ dysfunction, especially in kidneys, where its accumulation augments the pre-existent tubular and vascular injury, resulting in the delay of the renal recovery. Uremic toxins are the central actors in multi-organ breakdown and they negatively affect the renal recovery after acute injury. Thereby, research on the optimal renal replacement therapy cartridge that is able to clear specific toxins or pharmacological therapies with the effect of bacteria metabolites strongly requires further exploration.

### 2.3. Uremic Toxins in Kidney Transplantation

Kidney transplantation represents one of the most effective treatments for patients with end-stage renal disease, since it significantly improves the survival rate and ameliorates the quality of life. Moreover, the implementation of immunosuppressive therapies was shown to reduce acute rejection episodes and increase the organ survival. On the other hand, it leads to an increase in complications related to the reduced immunocompetence of transplanted patients, such as infections, with the subsequent requirement of antimicrobial therapy. Kidney transplantations are often associated with infectious complications, which increase the mortality rate of recipients. Over the previous years it has been demonstrated that kidney transplantation induces perturbations in the gastrointestinal flora that could act as key players in transplant-associated infections [110]. Moreover, gut dysbiosis can influence the immune system of the recipient and recent evidence has highlighted a relationship between alterations in gastrointestinal communities and poor outcomes in renal transplant recipients [111]. Although it remains to be clarified whether this condition of gut dysbiosis is strictly related to kidney transplantation or is a common element for all patients with end-stage renal disease, it has been reported that substantial differences characterize the microbial pattern of recipients in comparison with healthy subjects [29,111]. In particular, it has been shown that the gut microbial populations of kidney transplant recipients are characterized by the prevalence of *Firmicutes*, whereas an increase in *Proteobacteria* was detected within fifteen days after transplantation [29]. Gut dysbiosis represents a risk factor for the optimal functioning of renal graft and can adversely affect the outcome of a kidney transplantation through alterations of both the host’s immune system and inflammatory cytokines production [111]. Indeed, gut dysbiosis can be associated with an impairment of the gastrointestinal barrier integrity, which can cause bacterial displacement into the systemic circulation that triggers the pro-inflammatory response. These conditions can cause graft inflammation and, in the end, graft rejection via the autoreactive and alloreactive lymphocyte [111,112]. Microbial metabolism is essential in producing uraemic retention solutes, such as PBUTs. The toxicity of PBUTs has been well-documented in a plethora of pathophysiological mechanisms in kidney disease [113,114,115]. However, the impact of renal transplantation on these toxins has not been completely explored so far. Liabeuf et al. reported that the IS amount is markedly lower in transplanted recipients after 12 months than in non-transplanted CKD subjects with similarly estimated glomerular filtration rates [116]. Additionally, kidney transplantation strongly lowered the blood levels in a large amount of PBUTs, including phenols, and to a smaller degree, indoles [117]. Several other uremic compound such as HA, PHS, IAA, and kynurenines, have also been described as significantly reduced in the first week after transplantation [118], although their reduction seems to not be correlated with the amelioration of neurocognitive functions. Taken together, these results demonstrated that kidney transplantation can affect uremic toxins levels, since their accumulation largely declined after renal engraftment. Thus, it could be speculated that the transplant itself is able to influence the microbiome diversity, the leaky gut, and consequently, the adsorption of such toxins [117]. This condition could be dependent on immunosuppression and prophylactic antimicrobial therapy with the consequent reduction and/or alteration of PBUTs production.

Microbiota-derived metabolites may have a negative impact on the outcomes of graft survival and function, since high levels of PCS and IS can cause the production of pro-fibrotic molecules and inflammatory cytokines from renal tubular cells, resulting in increased tubulointerstitial fibrosis, cellular injury, and nephrotoxicity [58,119]. Korytowska et al. in 2021 demonstrated that salivary IS can be employed as a non-invasive diagnostic marker in order to recognize the loss/deterioration of the graft function (DoGF) more than a year following kidney transplantation [120]. The study was carried out on 92 kidney transplant recipients and, although it presents some limitations to the proposed model, this study assessed the role of IS as a potential predictor of DoGF and as a useful marker to prevent graft failure, and therefore, extending the survival and the functioning of the transplanted kidney [120]. Nevertheless, in the case of some uremic toxins, different and contrasting results, with respect to those previously described, have been reported. For example, levels of middle-molecular uremic toxin fibroblast growth factor 23 (FGF23) remain high even after kidney transplantation [121]; alternatively, plasma levels of the small molecule asymmetric dimethylarginine (ADMA) increase immediately after kidney transplantation, and its levels reduce over the weeks without this being reflected in an improvement of renal graft function [122]. The understanding of the biological mechanisms underlying these findings is still partial, partly because of the limited number of studies carried out so far on this topic. Thus, it is clear that these results highlight the need for further investigation of how uremic toxins may affect renal transplantation and vice versa, with the aim of implementing specific therapeutic interventions to improve the outcome of kidney transplantations.

## 3. FMT

In recent years, FMT has been acknowledged as an impactful strategy to manipulate the gut microbiome [10]. The first report of an FMT application derives from ancient Chinese medicine for treating severe diarrhea, while its first clinical employment was documented in the 20th century for the management of pseudomembranous colitis [123]. Currently, FMT represents the first-line strategy for resolving recurrent *Clostridium difficile* infection (rCDI). Similarly to solid organ transplantation, the conceptualization behind the FMT procedure is based on the replacement of a dysbiotic flora with a healthy microbiome. In clinical practice, FMT concerns the inoculation of fecal material from a healthy subject in the diseased gut of a recipient patient. Hence, the administration of a healthy microbiomes purposes is to decolonize and repopulate the intestinal environment with a stable microbial community [124]. Based on this evidence, fecal transplantation could represent a novel and promising treatment for different dysbiosis-associated disorders.

### FMT Procedure

Similarly to solid organ transplantation, the fecal transfer procedure demands stool collection from healthy donors [125]. Importantly, the workflow concerning donor eligibility is based on rigorous hematological and stool analysis to avoid the transfer of transmittable diseases such as HIV, Hepatitis, syphilis, *C. difficile*, protozoa, and helminths [126]. Additionally, potential donors undergo a medical interview to check for any history of infectious diseases or gastrointestinal and metabolic disorders. Of note, following the donation, the collected stool can be either directly transplanted into the recipient or frozen for later use. As stated in the European guidelines for FMT application, different routes can be employed for fecal transfer [126]. Depending on the GI-tract site, the healthy microbiota can be conducted by lower (colonoscopy or rectal enema) or upper (naso-gastric or nasojejunal) route [127]. Widely employed, the colonoscopy demonstrated a success rate of 90% for resolving rCDI. On the other hand, fecal administration by enema is employed only when colonoscopy is inaccessible. Finally, due to the high risks of tissue perforation, the upper GI-tract route is hardly ever used [127]. Interestingly, in the view of improving invasiveness and patient compliance, a novel capsule-mediated FMT approach was developed in recent years for treating recurrent *C. difficile* infections [128,129]. Notably, the so-called oral FMT is based on the encapsulation of lyophilized microbiota into a 0/00 gastro-resistant capsule, which excellently vehiculates the healthy-associated flora into the recipient’s intestine [130]. Clinical trials assessed the effectiveness of oral fecal transplants for resolving rCDI, with a success rate of 90% associated with lower unfavorable events [131,132,133]. Moreover, as reported by a few meta-analyses, the capsulo-mediated FMT strongly improved the microbiota tolerability compared with the traditional routes of delivery [131].

## 4. FMT and Kidney Disease

**Figure 2:**
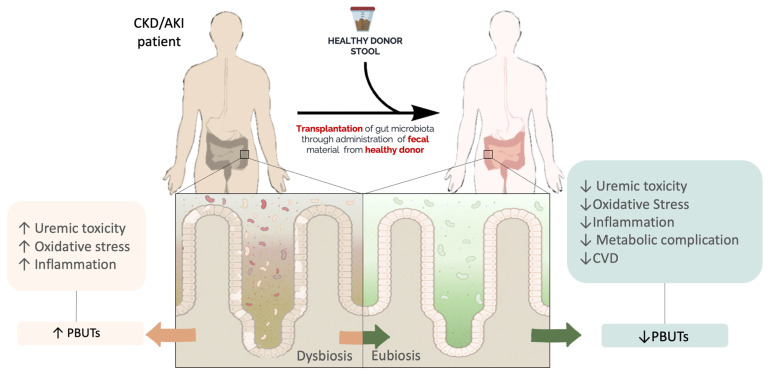
The potential effect of FMT on kidney disease. Abbreviations: CKD, chronic kidney disease; AKI, acute kidney injury; PBUTs: protein-bound uremic toxins; an CVD, cardiovascular disease.

Although FMT exerts a primary function in the modulation of the gastrointestinal flora, its beneficial effects extend to a systemic level in regulating inflammation, oxidative stress, and metabolic disorders. Based on this evidence, in recent years, the application of FMT was implemented in many dysbiosis-associated diseases but also extra-intestinal disorders, such as metabolic syndrome and neurodegenerative diseases [10]. Recently, the mounting data have highlighted the detrimental effects of dysbiosis-associated uremic toxicity in kidney disease. In these settings, a broad number of strategies focused on gut microbiota modulation, such as biotic supplementation, have demonstrated positive results in lowering the circulating levels of PBUTs. On the other hand, a substantial amount of data produced contradictory findings [134]. Furthermore, despite their time-limited effect, the strategies based on pre-, pro-, and syn-biotics provide early evidence that the whole microbiota repopulation may exert a long-term effect. Thus, the FMT could represent an auspicious strategy in preserving renal injury from the deleterious effects of gut-derived PBUTs (Figure 2). Research is in its infancy and prior to the application of FMT on patients, researchers are focusing on in vivo studies.

Originally, the fecal transplantation procedure was applied to elucidate the role of intestinal dysbiosis in kidney diseases (Table 2). Uchiyama et al. elegantly demonstrated that the transplantation of CKD-derived microbiomes reproduced the uremic phenotype in healthy, germ-free mice. After the fecal transplantation, the recipient mice showed a profound dysbiosis associated with high levels of circulating uremic toxins, including PHS, IS, and HA. Moreover, the overproduction of such toxins induced sarcopenia, insulin resistance, and intestinal permeability in healthy recipient mice [85]. Conforming with these data, Li and co-workers demonstrated that the fecal contents of streptozotocin-treated DN mice induced a microbiome alteration associated with high levels of TMAO and LPS in antibiotic-treated recipients. The recipient mice displayed a high grade of renal damage correlated with elevated levels of bacterial toxins when transplanted with the fecal contents of DN mice with severe proteinuria (≥300 mg/24 h). On the other hand, low-grade injury associated with a lower concentration of TMAO and LPS was induced when recipients were transplanted with fecal microbiota from mice with moderate proteinuria (<300 mg/24 h) [24]. Notably, a disease-associated microbial pattern was also detected between the two groups of DN mice, indicating that the dissimilarity in microbiome patterns can influence the different outcomes of DN.

Comparably, Wang and collaborators evaluated that the fecal microbiota of individuals with advanced CKD worsened the renal injury when transferred in CKD rodents. The authors show that the dominance of several bacteria, including *Eggarthella lenta* and *Fusobacterium nucleatum,* was closely related to the increase in many PBUTs, such as HA, PHS, PCS, and IS. Furthermore, the transplantation of healthy donor stool in diseased rodents improved the circulating level of uremic solutes, such as creatinine, urea, and PBUTs [14]. Yang and colleagues further explored the relationship between microbiota and AKI by using the microbiota transplantation procedure. Specifically, they observed that the post-AKI microbiome strongly influenced the severity of the ischemia/reperfusion injury when administered in germ-free mice. Altogether, these findings corroborated the causal link between the detrimental microbiome and kidney disease [27].

In the last two years, few in vivo studies have explored the implementation of FMT in renal disease, supporting the therapeutic contributions of microbiota replacement in uremia (Table 2). These attempts aimed to correct PBUTs levels, inflammation, and metabolic dysfunction. Liu et al. explored the effects of FMT on 1/2 nephrectomy-induced CKD rats using fecal contents from sham-operated donors. Of note, before the transplantation, the CKD group was sterilized by an antibiotic cocktail in order to deplete the resident flora. Fecal administration restored the gut eubiosis of CKD rodents by affecting the enrichment of *Lactobacillaceae* (*L. johnsonii* and *L. intestinalis*). Interestingly, the FMT strongly lowered the blood levels of a large amount of PBUTs, including IS, PCS, PHS, TMAO, and Phenylacetyl glycine. In addition, the sham-related microbiome improved renal injuries and the inflammatory status when transplanted in CKD animals [135]. A similar study was performed by Barba et colleagues using adenine-induced CKD mice. In this study, it was mainly observed that the transfer of healthy mice microbiota in CKD recipients was able to correct the uremic dysbiosis, resulting in the reduction of plasma tyrosine-derived PBUTs. Moreover, the improvement of glucose tolerance and IR was further observed after three incidences of FMT administrations. These findings suggest that the reduction of PCS and PCG was effective in ameliorating CKD-related metabolic complications [136].

Promising results have also been found in models of DN rodents treated with healthy microbiota. In this context, the investigation by Hu et al. evidenced that the repopulation of dysbiotic microbiota from healthy donor GI-flora improved the tubular lesion by ameliorating the apoptosis of TECs in DN rats. Furthermore, FMT enriched the amount of acetate-producing *Prevotellaceae*, *Ruminococcaceae*, and *Lactobacillaceae* families, leading to the correction of cholesterol homeostasis. Finally, the decrease in serum IL-6 suggested an improvement in systemic inflammation [137]. In a similar fashion, Lu et al. demonstrated that DN rats that were transplanted with healthy flora normalized the altered insulin pathway in podocytes by reducing the circulating levels of microbiota-derived acetate. Acetate reduction was also associated with GPR43 downregulation in podocytes. Curiously, FMT was also able to recover both the morphology and number of podocytes [138]. More recently, an in vivo study found that the replacement of dysbiotic microbiota with healthy communities significantly ameliorated the metabolic complications associated with diabetic nephropathy in leptin-deficient BTBR mice. The fecal bacteriotherapy downregulated the expression of TNF-α in enterocytes and reestablished gut permeability [139]. Despite the lack of PUBTs analysis in these studies, it can be speculated that the recovery of metabolic complications may be associated with a decrease in several PBUTs, including PCS, PCG, and IS, due to their involvement in metabolic dysfunction. The beneficial effects of fecal transplantation were further explored in IgA nephropathy, where dysbiosis is involved in driving its pathogenesis by influencing the host immune response [140]. In this scenario, Lauriero and collaborators demonstrated that the graft of healthy human microbiota mitigated inflammation and improved kidney injury and glucose tolerance in a mice model of IgAN. Additionally, when transplanted, the healthy microflora reversed the accumulation of dysbiosis-related cresols and indoles and intensified the gut production of several SCFAs [140].

On the other hand, despite the evidence concerning the beneficial effects of fecal bacteriotherapy on CKD, there is a limited number of data that support the implementation of FMT in AKI. Emal et al. detected that the depletion of the intestinal flora via antibiotic treatment was able to attenuate kidney injury in the I/R model of AKI. Furthermore, when compared with the antibiotic-untreated group, the sterilized mice exhibited an improvement in renal dysfunction related to the reduction of several pro-inflammatory factors (i.e., TNF, IL-6, MCP-1, and MIP). Additionally, the gavage of fecal contents from untreated donors restored the granulocyte influx and the expression of chemokine receptors in resident macrophages. However, they further reported that FMT did not influence the renal function of AKI recipients [141]. In contrast to this observation, the study by Nakade et al. indicated that the treatment of healthy microbiota for twelve weeks significantly alleviated acute renal injury and intestinal dysfunction [142].

Finally, within the field of kidney disease, the clinical use of FMT was documented only in two different case reports regarding CKD patients with intestinal discomfort (Table 2). In the investigation by Zhao et al., the 20-week fecal transplantation from healthy donors to IgAN recipients significantly lowered the proteinuria and alleviated intestinal distress by improving α and β diversity [143]. Consistent with this finding, Zhou et colleagues reported that FMT decreased the levels of small water-soluble toxins and reversed intestinal edema and diarrhea in a patient suffering from membranous nephropathy [144]. The oral FMT treatment, using encapsulated fecal microbiota derived from a healthy gut, was used to treat a patient with Focal Segmental Glomerulosclerosis. The administration of twenty capsules once a week for a total of three weeks was able to refurbish the lipidic profile and the relative proportion of various microbial families, including *Prevotellaceae* and *Bacteroidaceae*. Moreover, authors showed a reduction in several proinflammatory mediators [145].

Traditionally, fecal transplantation is performed using feces collected by untreated healthy donors (Table 2). However, a small group of studies showed that the stimulation of donor microbiota with biotic supplements before transplantation could represent an encouraging strategy in augmenting the effect of FMT. For example, the fecal contents of resveratrol-treated rodents demolished the elevated ratio of PBUTs producing bacteria, including *Firmicutes*, *Tenericutes*, *Deferribacteres*, and *Enterococci*, when implanted in CKD recipients. The manipulated microbiota was able to reverse the gut injury and inflammation in diseased rodents [146]. In another study, healthy mice treated with the probiotic *Astragalus membranaceus* were used as donors of fecal microbiota. When transplanted, the *A. membranaceus*-supplemented microbiota strongly improved the intestinal permeability and increased the proportion of *Akkermansia* and *Lactobacillus* in CKD mice. Moreover, the reinforced microbiome alleviated glomerular dysfunction and tubular fibrosis [147]. Despite the fact that the levels of PBUTs were not reported, it can be hypothesized that the demolition of gut dysbiosis together with the recovery of the intestinal barrier may have limited their blood accumulation. Finally, Zheng et al. developed an engineered microbiota by micro-encapsulating a synthetic cocktail of different bacteria. Interestingly, they reported that its administration markedly lowered several small solutes, including urea and creatinine, in both mice and swine models of AKI and CKD [148]. Altogether, these data lay the foundation for promising “reinforced FMT” approaches which could represent a new frontier in personalized therapies to modulate the gut microbiome.

**Table 2:** Summary of in vivo studies based on the application of fecal transplantation in kidney disease.

Author, Year, Ref	AIM	FMT Modality	Experimental Design for FMT Procedure	Main Findings	Microbiome Evaluation	Effect on Uremic Toxins
Uchiyama et al., 2020 [85]	Explore the role of uremic dysbiosis in CKD-associated IR and sarcopenia.	**Donor:** CKD mice treated with adenine diet (0.2%). **Recipient:** healthy germ-free mice.	FMT was performed once in healthy germ-free mice from control mice or CKD mice.	Uremic flora induced sarcopenia, IR, and intestinal permeability in recipient mice.	Decrease in *Bacilli, Lactobacillales,* and *Lactonifactor.* Increase in *Erysipelotrichi, Erysipelotrichales Allobaculum, Clostridium,* and *Alistipes.*	↑ IS ↑ PHS ↑ HA ↑ IL-6
Li et al., 2020 [24]	Explore the role of gut microbiota in diabetic nephropathy.	**Donors:** DN mice (STZ-treated) grouped by severe or moderate proteinuria. **Recipient:** Antibiotic-treated mice.	Study 1: mice were treated with antibiotics and then with FMT from severe/mild proteinuria group, daily, for 3 days. After, FMT mice were treated with STZ. Study 2: STZ was administered before FMT in antibiotic-treated mice. FMT was performed from severe/mild proteinuria group, daily, for 3 days.	Alterations in gut microbiome modulated the kidney function of DN models. The author suggests that *Allobaculum* and *Anaerosporobacter* may worsen renal function, while *Blautia* may be a protective factor in DN.	*Firmicutes* were more abundant in mice treated with fecal content of mild proteinuria. *Allobaculum* increased in the recipients transplanted with severe proteinuria flora. *Blautia* increased in mice that received the microbiome from the mild proteinuria mice.	FMT from severe proteinuria mice: ↑ TMAO ↑ LPS ↓ SCFAS FMT from moderate proteinuria mice: ↓ TMAO ↓ LPS ↑ SCFAS
Wang et al., 2020 [14]	Explore the relationships between gut flora and renal failure.	**Donor:** ESRD patients or healthy donors. **Recipient:** CKD mice (adenine treated) and CKD rats (5/6 nephrectomy treated with antibiotics).	CKD mice: 200 ul (0.1 g/mL) of pooled stool was gavaged for 3 days. CKD rats: 1 mL (0.1 g/mL) of pooled stool was gavaged daily for 3 weeks.	FMT from ESRD patients increased uremic toxins levels and aggravated kidney injury. FMT from healthy donors lowered serum creatinine, urea, and several uremic toxins.	*E. lenta* was found to increase the production of HA and PAG. *Fusobacterium nucleatum* increased the production of indole and phenol.	Mice/rats receiving ESRD stool: ↑ IS ↑ pCS ↑ PAG ↑ PhS ice/rats receiving healthy donor stool: ↓ TGF-β1
Yang et al., 2020 [27]	Explore the link between kidney and gut microbiota during AKI.	**Donor:** sham-operated mice or IRI mice models. **Recipient:** germ-free mice.	FMT was performed via gastric gavage on day 0 and day 10.	After renal IRI, gut microbiota modulated inflammation and severity of kidney injury.	NA	↓ TNF-α ↓ IFN-γ
Liu et al., 2022 [135]	Explore the role of FMT on CKD.	**Donor:** sham-operated rats. **Recipients:** 1/2 nephrectomy rats treated with an antibiotic cocktail.	FMT was performed daily for 21 days.	FMT improved kidney function and oxidative stress.	FMT restored the proportion of *Lactobacillus johnsonii* and *Lactobacillus intestinalis.*	↓ IS ↓ PCS ↓ PhS ↓ Phenylacetyl glycine ↓ TMAO
Barba et al., 2020 [136]	Explore the role of FMT on the metabolic complication and uremic toxins level.	**Donor:** healthy mice. **Recipient:** CKD mice induced with adenine diet (0.25%).	FMT was performed once a week for a total of three weeks by oral gavage.	FMT improved glucose intolerance, and IR.	FMT induced a significant amelioration α-diversity and restored the abundance of *Oscillospira* and *Desulfovibrio.*	↓ PCS ↓ PCG
Hu et al., 2020 [137]	Investigate the role of microbiome on diabetic nephropathy.	**Donor:** healthy rats. **Recipient:** DN rats (STZ-treated rat).	FMT was performed once a day for 3 days.	FMT improved tubulointerstitial injury and inflammation and reduced both triglycerides and serum acetate levels.	FMT restored the proportion of *Prevotellaceae*, *Ruminococcaceae,* and *Lactobacillaceae.*	↓ IL-6
Lu et al., 2021 [138]	Investigate the role of gut microbiota in diabetic nephropathy.	**Donor:** healthy rats. **Recipient:** STZ-induced rat model of DKD.	Total of 200 uL of the suspended fecal microbiota was administered in diabetic rats by oral gavage.	FMT improved renal injury, reduced the serum acetate levels, and restored renal insulin signaling via Akt phosphorylation.	NA	NA
Bastos et al., 2022 [139]	Investigate the efficacy of FMT in a model of type DKD using BTBR^ob/ob^ mice.	**Donor:** BTBR wild-type mice. **Recipient:** BTBR^ob/ob^ mice: homozygous for the leptin gene knockout.	FMT was administered via rectal delivery.	FMT improved body weight and glomerular hypertension and reversed IR and colon permeability.	The treatment enriched the abundance of *Odoribacteraceae.*	↓ TNF-α
Lauriero et al., 2021 [140]	Explore the link between gut flora and IgA nephropathy outcome.	**Donors:** healthy controls or non-progressor IgAN or progressor IgAN patients. **Recipient:** antibiotic-treated humanized IgAN mice.	FMT was performed for five days.	FMT modulated renal phenotype together with BAFF levels, IR, and inflammation.	FMT modulated the proportion of *Bacteroidetes*, *Bacteroides* spp., and *Actinobacteria,* and increased colonization of *Firmicutes*.	↓ indole ↓ pCS ↓KC
Emal et al., 2017 [141]	Investigate the role of gut microbiota in kidney disease.	**Donor:** untreated mouse. **Recipient:** AKI mouse (antibiotic-treated before I/R injury).	FMT was performed for three days via oral gavage.	FMT modulated the expression of macrophage influx and the expression of chemokines receptors.	NA	NA
Nakade et al., 2018 [142]	Explore the pathophysiologic role of microbiota associated with D–amino acids AKI.	**Donor:** healthy B6 mouse. **Recipient:** germ-free B6 AKI mouse.	FMT was performed for 12 weeks before I/R injury via rectal route.	FMT protected against tubular injury in AKI mouse.	NA	NA
Case reports:						
Zhao et al., 2021 [143]	Case report of FMT treatment in two female patients with IgA nephropathy.	**Donor:** male healthy donor. **Recipient:** female patients with IgAN with intense GI discomfort.	FMT was performed 40 times (200 mL/day for 5 days/week) and then a further 57 times over 5 months.	FMT lowered the 24 h urinary protein and improved the protein loss.	Case 1: FMT reversed α and β diversity. Case 2: FMT decreased *Verrucomicrobia.*	NA
Zhou et al., 2021 [144]	Case report of FMT treatment in an adult patient with membranous nephropathy (MN).	**Donor:** male healthy donor. **Recipient:** patient with MN.	FMT was performed 2 times: on day 0 and after 28 days.	FMT decreased urea and creatine levels and reversed the symptoms of edema and diarrhea.	NA	NA
Zhi et al., 2022 [145]	Evaluate the effect of oral FMT (encapsulated FMT) on Focal Segmental Glomerulosclerosis.	**Donor:** healthy donor. **Recipient:** CKD patient.	Twenty FMT capsules were administered once a week for three weeks via oral capsule.	FMT ameliorated urinary proteinuria and hyperlipidemia (triglyceride and cholesterol levels). A reduced levels of proinflammatory mediators was observed in the first three month after therapy.	FMT treatment restored the balance of *Prevotella coprii* and *Bacteroides uniformiis.*	↓ IL-5 ↓ IL-4 ↓ IL-1β
FMT with pre-stimulated donors:						
Cai et al., 2020 [146]	Determine the role of gut microbiome and resveratrol on diabetic nephropathy.	**Donor:** resveratrol-treated mice or control db/m. **Recipient:** animal model of DN (db/db mice).	FMT was performed by oral gavage daily for 7 days.	FMT improved renal dysfunction, intestinal permeability, and inflammation.	FMT increased *Proteobacteria*, *Alistipes, Turicibacter, Odoribacter,* and *Rikenellagenus* and reduced the abundance of *Firmicutes, Tenericutes, Deferribacteres,* and *Enterococci.*	↓ TNF-α, ↓ IFN-γ, ↓ IL-6, ↓ IL-1β
Han et al., 2021 [147]	Explore the protective effect of microbiome and *Astragulis membranaceus* on CKD.	**Donor:** CKD mice treated with Astragalus membranaceus. **Recipient:** CKD mice (Cyclosporin A-treated).	FMT was performed for 6 weeks.	FMT ameliorated kidney function (glomerular dysfunction, renal tubules vacuolization, and fibrosis).	FMT reversed the proportion of *Akkermansia* and *Lactobacillus.*	NA

Abbreviations: IR, insulin resistance; CKD, chronic kidney disease; AKI, acute kidney injury; IS, Indoxyl sulfate; PHS, phenyl sulphate; HA, hippuric acid; IL; DN, diabetic mephropathy; DKD, diabetic kidney disease; STZ, streptozotocin; TMAO, Trimethylamine N-Oxide, lipopolysaccharide; SCFAs, short chain fatty acids; ESRD, end-stage renal disease; PAG, phenylacetylglutamine; PCS, P-cresyl sulfate; TGF-β1, transforming grow factor β1; IRI, ischemia reperfusion injury; TNF-α; IFN-γ, interferon-γ; PCG, p-cresyl glucuronide; BTBR^ob/ob^ mice, black and tan brachyuric obese mutant mice; IgAN, IgA nephropathy; BAFF, B cells’ activating factor; GI, gastrointestinal; MN, membranous nephropathy; and NA, not assessed.

### Effects of FMT on Kidney Transplantation

The relevant data support the interconnection between gut microbial profiles and the outcomes in renal transplantation [149]. Great amounts of research have highlighted that the microflora alteration is induced by the elevated administration of immunosuppressants and antimicrobial-prophylactic drugs to prevent graft failure and infections. The detrimental microflora is usually marked by the alteration of an alpha/beta diversity correlated with high amounts of *Proteobacteria* [28,29,150,151]. The microbiota profile alters the host’s immune response through the activation of numerous pivotal factors (i.e., Myd-88 or TLR-9) and affects the over-proliferation of many lymphocyte populations, worsening the functional outcome of the graft [112,152]. Another important aspect is related to the link existing between dysbiosis and infectious complications [111]. Magruder et al. demonstrated that dysbiosis could be connected with the occurrence of a urinary tract infection (UTI) in transplanted patients [153]. As mentioned previously, the evaluation of metabolic profiles has been considered a hallmark of bacterial richness and diversity [149]. Poesen and colleagues observed that PBUTs derived from kidney-transplanted patients were reduced in patients with CKD [117]. This finding was further sustained by the evidence that the changes in severity of gut bacteria was achieved immediately after transplantation and ameliorated after twelve months after transplantation [154]. The accumulated knowledge supports the notion that diet and biotic supplements could represent therapeutic approaches in modulating the GI microflora and ameliorating the outcome of the graft. Until now, there have been limited data concerning the efficacy of fecal transplantation in preventing the accumulation of PBUTs. Stripling et al. reported the effectiveness of fecal bacteriotherapy in a kidney recipient with rCDI [155]. In another study, the authors demonstrated that FMT led to the resolution of recurrent CDI in eight patients who were non-transplant [156].

## 5. FMT: A Mixed Blessing for Kidney Disease

Intestinal dysbiosis is closely linked to kidney disease. Moreover, the high accumulation of a large amount of microbiota-derived PBUTs is strongly associated with metabolic dysfunction, inflammation, and CVI [85,87,157]. In recent years, the use of oral supplements together with different dialysis settings has been designed to counteract the accumulation of uremic solutes. However, the effectiveness of these strategies remains controversial [134]. Beyond the lowering of uremic toxins, strong evidence supports the pivotal impact of FMT in restoring uremia-related complications by ameliorating the gut uremic milieu. Moreover, in many pathologic conditions, fecal transplantation was related to the improvement of bacterial richness [158,159,160]. In line with these data, the application of FMT in kidney disease indicates that the gut recolonization with a healthy microbiome is able to re-establish a stable microbial community in the gut of the recipient [135,136,140]. Moreover, as a result of FMT, a strong reduction of many PBUTs was observed, especially those derived from the proteolytic metabolism of tyrosine, tryptophan, and choline [135,136,140]. Based on this observation, it can be assumed that gut repopulation could alleviate the detrimental effect of uremic toxins.

In recent years, a large number of data demonstrated that that fecal transplantation is able to induce recipient homeostasis by modulating inflammation and metabolic dysfunction [126,161]. In CKD, the retention of PBUTs is associated with a wide range of metabolic disorders including insulin resistance and dyslipidemia [85]. Interestingly, the data presented in this study show that fecal transplantation may represent a novel approach to restore glucose intolerance, insulin signaling, and triglyceride and cholesterol levels. Moreover, FMT ameliorated the systemic inflammation in CKD and AKI recipients, since a reduction of many pro-inflammatory mediators (e.g., IFN-γ, IL-6, TNF-α, and IL-1β) was observed after the treatment [27,135,145,146]. Comparable results were observed in many other diseases in which the fecal transplantation alleviated inflammation and the immune response [161,162,163]. Hence, the loss of pathogenic flora followed by the reduction of several microbial toxins such as LPS and PBUTs may clarify, at least in part, the modulation of the local immune system leading to the downregulation of inflammation.

Finally, several data show that the gut dysbiosis represents a pivotal mechanism in modulating the renin-angiotensin-aldosterone system (RAAS) in kidney disease [164]. In detail, few pieces of evidence suggest that the RAAS activation could be, at least in part, linked to the microbiota-derived PBUTs. Consistently with this statement, experimental studies demonstrated that the dysbiosis-related uremic toxins are involved in promoting the kidney injury by activating the intrarenal angiotensin II [165,166]. Based on this evidence, the improvement of RAAS after FMT may represent the missing link involved in the improvement of renal injury [77,135,139,142].

Despite its growing experimentation in animal models of kidney disease, there is no scientific guideline for the best approach. On the one hand, various procedures in fecal contents processing, storage, and delivery, are not well standardized. Additionally, the frequency and duration of FMT administration can profoundly affect FMT results. Finally, the use of antibiotics before transplantation to deplete the dysbiotic flora remains controversial. Of note, particular focus should be placed on the experimental model of kidney disease including adenine-treated mice, 5/6 nephrectomy.

## 6. Conclusions

In recent years, FMT has been accepted as an impactful strategy to manipulate the gut microbiome. Currently, FMT represents a first-line therapy for resolving rCDI. *C. difficile* fecal transplantation is demonstrated to be an auspicious strategy in treating a great number of disorders, including kidney disease. In this scenario, several in vivo studies shed light on FMT applications in the context of CKD as a novel strategy for regulating PUBUT levels. Although it produced promising results, the method’s standardization including the delivery route, fecal amount, and the time of administration, should be well-established before human application. From a practical standpoint, for the treatment of several dysbiosis-associated chronic diseases, the FMT probably needs to be administered over a long period. For instance, in CKD, where dysbiosis represents a result of chronic renal injury, a single FMT administration could be ineffective over the long term. Based on this observation, it can be hypothesized that capsulo-mediated FMT, due to its safety and low invasiveness, could represent the future direction to achieve “*chronic fecal microbiota repopulation*” therapy. Finally, the next generation of approaches could be represented by “reinforced FMT” therapies to personalize the microbiome interventions.

## Figures and Tables

**Table 1:** Summary of microbially produced uremic toxins, class, precursor, property, and related toxic effect.

Name	Class	Precursor	Characteristics	Conventional HD Reduction Rate (%)	Effect of PBUTs	Ref
Indoxyl sulfate	Indoles	Tryptophan	PBUT	30%	CVD, vascular injury, bone disease, and nephrotoxicity	[46,47,48,49]
Indole3 Acetic Acid	Indoles	Tryptophan	PBUT	40%	Cardiovascular dysfunction, endothelial damage, and cognitive impairment	[50,51,52]
Kynurenine, kynurenic acid, quinolinic acid	Kinurenine pathway	Tryptophan	PBUT	20%	Cardiovascular dysfunction and cognitive impairment	[53,54]
Hippuric Acid	Hippurates	Benzoic acid	PBUT	60–70%	Renal fibrosis and endothelial dysfunction	[55,56]
P-cresyl sulfate	Phenols	Tyrosine	PBUT	30%	Cardiovascular damage, renal tubular injury, and insulin resistance	[57,58,59]
P-cresyl glucuronide	Phenols	Tyrosine	PBUT	70%	Vascular damage	[60]
TMAO	Amine oxide	Choline, betaine, carnitine	Water-soluble compound	80%	CVD and renal inflammation	[61,62,63]

Abbreviations: HD, hemodialysis; PBUTs, protein-bound uremic toxins; CVD, cardiovascular disease.

## Data Availability

Not applicable.

## References

[1] Rinninella E., Raoul P., Cintoni M., Franceschi F., Miggiano G., Gasbarrini A., Mele M. (2019). What Is the Healthy Gut Microbiota Composition? A Changing Ecosystem across Age, Environment, Diet, and Diseases. Microorganisms.

[2] Park J., Kato K., Murakami H., Hosomi K., Tanisawa K., Nakagata T., Ohno H., Konishi K., Kawashima H., Chen Y.-A. (2021). Comprehensive Analysis of Gut Microbiota of a Healthy Population and Covariates Affecting Microbial Variation in Two Large Japanese Cohorts. BMC Microbiol..

[3] King C.H., Desai H., Sylvetsky A.C., LoTempio J., Ayanyan S., Carrie J., Crandall K.A., Fochtman B.C., Gasparyan L., Gulzar N. (2019). Baseline Human Gut Microbiota Profile in Healthy People and Standard Reporting Template. PLoS ONE.

[4] Manor O., Dai C.L., Kornilov S.A., Smith B., Price N.D., Lovejoy J.C., Gibbons S.M., Magis A.T. (2020). Health and Disease Markers Correlate with Gut Microbiome Composition across Thousands of People. Nat. Commun..

[5] Arumugam M., Raes J., Pelletier E., le Paslier D., Yamada T., Mende D.R., Fernandes G.R., Tap J., Bruls T., Batto J.-M. (2011). Enterotypes of the Human Gut Microbiome. Nature.

[6] Palmas V., Pisanu S., Madau V., Casula E., Deledda A., Cusano R., Uva P., Loviselli A., Velluzzi F., Manzin A. (2022). Gut Microbiota Markers and Dietary Habits Associated with Extreme Longevity in Healthy Sardinian Centenarians. Nutrients.

[7] Larsen N., Vogensen F.K., van den Berg F.W.J., Nielsen D.S., Andreasen A.S., Pedersen B.K., Al-Soud W.A., Sørensen S.J., Hansen L.H., Jakobsen M. (2010). Gut Microbiota in Human Adults with Type 2 Diabetes Differs from Non-Diabetic Adults. PLoS ONE.

[8] Ott S.J. (2004). Reduction in Diversity of the Colonic Mucosa Associated Bacterial Microflora in Patients with Active Inflammatory Bowel Disease. Gut.

[9] Wells P.M., Adebayo A.S., Bowyer R.C.E., Freidin M.B., Finckh A., Strowig T., Lesker T.R., Alpizar-Rodriguez D., Gilbert B., Kirkham B. (2020). Associations between Gut Microbiota and Genetic Risk for Rheumatoid Arthritis in the Absence of Disease: A Cross-Sectional Study. Lancet Rheumatol..

[10] Vijay A., Valdes A.M. (2022). Role of the Gut Microbiome in Chronic Diseases: A Narrative Review. Eur. J. Clin. Nutr..

[11] Al Khodor S., Shatat I.F. (2017). Gut Microbiome and Kidney Disease: A Bidirectional Relationship. Pediatr. Nephrol..

[12] Wu I.-W., Gao S.-S., Chou H.-C., Yang H.-Y., Chang L.-C., Kuo Y.-L., Dinh M.C.V., Chung W.-H., Yang C.-W., Lai H.-C. (2020). Integrative Metagenomic and Metabolomic Analyses Reveal Severity-Specific Signatures of Gut Microbiota in Chronic Kidney Disease. Theranostics.

[13] Vaziri N.D., Wong J., Pahl M., Piceno Y.M., Yuan J., DeSantis T.Z., Ni Z., Nguyen T.-H., Andersen G.L. (2013). Chronic Kidney Disease Alters Intestinal Microbial Flora. Kidney Int..

[14] Wang X., Yang S., Li S., Zhao L., Hao Y., Qin J., Zhang L., Zhang C., Bian W., Zuo L. (2020). Aberrant Gut Microbiota Alters Host Metabolome and Impacts Renal Failure in Humans and Rodents. Gut.

[15] Wong J., Piceno Y.M., DeSantis T.Z., Pahl M., Andersen G.L., Vaziri N.D. (2014). Expansion of Urease- and Uricase-Containing, Indole- and p-Cresol-Forming and Contraction of Short-Chain Fatty Acid-Producing Intestinal Microbiota in ESRD. Am. J. Nephrol..

[16] Chen L., Chen D.-Q., Liu J.-R., Zhang J., Vaziri N.D., Zhuang S., Chen H., Feng Y.-L., Guo Y., Zhao Y.-Y. (2019). Unilateral Ureteral Obstruction Causes Gut Microbial Dysbiosis and Metabolome Disorders Contributing to Tubulointerstitial Fibrosis. Exp. Mol. Med..

[17] Gao B., Jose A., Alonzo-Palma N., Malik T., Shankaranarayanan D., Regunathan-Shenk R., Raj D.S. (2021). Butyrate Producing Microbiota Are Reduced in Chronic Kidney Diseases. Sci. Rep..

[18] Hu J., Zhong X., Yan J., Zhou D., Qin D., Xiao X., Zheng Y., Liu Y. (2020). High-Throughput Sequencing Analysis of Intestinal Flora Changes in ESRD and CKD Patients. BMC Nephrol..

[19] Shi K., Wang F., Jiang H., Liu H., Wei M., Wang Z., Xie L. (2014). Gut Bacterial Translocation May Aggravate Microinflammation in Hemodialysis Patients. Dig. Dis. Sci..

[20] Han S., Shang L., Lu Y., Wang Y. (2022). Gut Microbiome Characteristics in IgA Nephropathy: Qualitative and Quantitative Analysis from Observational Studies. Front. Cell Infect. Microbiol..

[21] De Angelis M., Montemurno E., Piccolo M., Vannini L., Lauriero G., Maranzano V., Gozzi G., Serrazanetti D., Dalfino G., Gobbetti M. (2014). Microbiota and Metabolome Associated with Immunoglobulin A Nephropathy (IgAN). PLoS ONE.

[22] Tao S., Li L., Li L., Liu Y., Ren Q., Shi M., Liu J., Jiang J., Ma H., Huang Z. (2019). Understanding the Gut–Kidney Axis among Biopsy-Proven Diabetic Nephropathy, Type 2 Diabetes Mellitus and Healthy Controls: An Analysis of the Gut Microbiota Composition. Acta Diabetol..

[23] Jiang S., Wang B., Sha T., Li X. (2020). Changes in the Intestinal Microbiota in Patients with Stage 5 Chronic Kidney Disease on a Low-Protein Diet and the Effects of Human to Rat Fecal Microbiota Transplantation. Med. Sci. Monit..

[24] Li Y., Su X., Gao Y., Lv C., Gao Z., Liu Y., Wang Y., Li S., Wang Z. (2020). The Potential Role of the Gut Microbiota in Modulating Renal Function in Experimental Diabetic Nephropathy Murine Models Established in Same Environment. Biochim. Biophys. Acta (BBA)—Mol. Basis Dis..

[25] Lei J., Xie Y., Sheng J., Song J. (2022). Intestinal Microbiota Dysbiosis in Acute Kidney Injury: Novel Insights into Mechanisms and Promising Therapeutic Strategies. Ren. Fail..

[26] Andrianova N.V., Popkov V.A., Klimenko N.S., Tyakht A.V., Baydakova G.V., Frolova O.Y., Zorova L.D., Pevzner I.B., Zorov D.B., Plotnikov E.Y. (2020). Microbiome-Metabolome Signature of Acute Kidney Injury. Metabolites.

[27] Yang J., Kim C.J., Go Y.S., Lee H.Y., Kim M.-G., Oh S.W., Cho W.Y., Im S.-H., Jo S.K. (2020). Intestinal Microbiota Control Acute Kidney Injury Severity by Immune Modulation. Kidney Int..

[28] Swarte J.C., Douwes R.M., Hu S., Vich Vila A., Eisenga M.F., van Londen M., Gomes-Neto A.W., Weersma R.K., Harmsen H.J.M., Bakker S.J.L. (2020). Characteristics and Dysbiosis of the Gut Microbiome in Renal Transplant Recipients. J. Clin. Med..

[29] Lee J.R., Muthukumar T., Dadhania D., Toussaint N.C., Ling L., Pamer E., Suthanthiran M. (2014). Gut Microbial Community Structure and Complications After Kidney Transplantation. Transplantation.

[30] Di Iorio B.R., Rocchetti M.T., de Angelis M., Cosola C., Marzocco S., di Micco L., di Bari I., Accetturo M., Vacca M., Gobbetti M. (2019). Nutritional Therapy Modulates Intestinal Microbiota and Reduces Serum Levels of Total and Free Indoxyl Sulfate and P-Cresyl Sulfate in Chronic Kidney Disease (Medika Study). J. Clin. Med..

[31] Bakhtiary M., Morvaridzadeh M., Agah S., Rahimlou M., Christopher E., Zadro J.R., Heshmati J. (2021). Effect of Probiotic, Prebiotic, and Synbiotic Supplementation on Cardiometabolic and Oxidative Stress Parameters in Patients With Chronic Kidney Disease: A Systematic Review and Meta-Analysis. Clin. Ther..

[32] Weiner D.E., Liu C.K., Miao S., Fielding R., Katzel L.I., Giffuni J., Well A., Seliger S.L. (2023). Effect of Long-Term Exercise Training on Physical Performance and Cardiorespiratory Function in Adults With CKD: A Randomized Controlled Trial. Am. J. Kidney Dis..

[33] Brookes E.M., Power D.A. (2022). Elevated Serum Urea-to-Creatinine Ratio Is Associated with Adverse Inpatient Clinical Outcomes in Non-End Stage Chronic Kidney Disease. Sci. Rep..

[34] Vaziri N.D., Yuan J., Norris K. (2013). Role of Urea in Intestinal Barrier Dysfunction and Disruption of Epithelial Tight Junction in Chronic Kidney Disease. Am. J. Nephrol..

[35] Vaziri N.D., Yuan J., Rahimi A., Ni Z., Said H., Subramanian V.S. (2012). Disintegration of Colonic Epithelial Tight Junction in Uremia: A Likely Cause of CKD-Associated Inflammation. Nephrol. Dial. Transplant..

[36] Wu T.-K., Lim P.-S., Jin J.-S., Wu M.-Y., Chen C.-H. (2018). Impaired Gut Epithelial Tight Junction Expression in Hemodialysis Patients Complicated with Intradialytic Hypotension. Biomed. Res. Int..

[37] Hsu H.-J., Yen C.-H., Wu I.-W., Hsu K.-H., Chen C.-K., Sun C.-Y., Chou C.-C., Chen C.-Y., Tsai C.-J., Wu M.-S. (2014). The Association of Uremic Toxins and Inflammation in Hemodialysis Patients. PLoS ONE.

[38] Vanholder R., Glorieux G., de Smet R., Lameire N. (2003). New Insights in Uremic Toxins. Kidney Int..

[39] Yamamoto S., Kazama J.J., Wakamatsu T., Takahashi Y., Kaneko Y., Goto S., Narita I. (2016). Removal of Uremic Toxins by Renal Replacement Therapies: A Review of Current Progress and Future Perspectives. Ren. Replace. Ther..

[40] Watanabe H., Noguchi T., Miyamoto Y., Kadowaki D., Kotani S., Nakajima M., Miyamura S., Ishima Y., Otagiri M., Maruyama T. (2012). Interaction between Two Sulfate-Conjugated Uremic Toxins, *p* -Cresyl Sulfate and Indoxyl Sulfate, during Binding with Human Serum Albumin. Drug Metab. Dispos..

[41] Mihaila S.M., Faria J., Stefens M.F.J., Stamatialis D., Verhaar M.C., Gerritsen K.G.F., Masereeuw R. (2020). Drugs Commonly Applied to Kidney Patients May Compromise Renal Tubular Uremic Toxins Excretion. Toxins.

[42] Lesaffer G., de Smet R., Lameire N., Dhondt A., Duym P., Vanholder R. (2000). Intradialytic Removal of Protein-Bound Uraemic Toxins: Role of Solute Characteristics and of Dialyser Membrane. Nephrol. Dial. Transplant..

[43] Itoh Y., Ezawa A., Kikuchi K., Tsuruta Y., Niwa T. (2012). Protein-Bound Uremic Toxins in Hemodialysis Patients Measured by Liquid Chromatography/Tandem Mass Spectrometry and Their Effects on Endothelial ROS Production. Anal. Bioanal. Chem..

[44] Basile C., Libutti P., di Turo A.L., Casino F.G., Vernaglione L., Tundo S., Maselli P., de Nicolo E.V., Ceci E., Teutonico A. (2011). Removal of Uraemic Retention Solutes in Standard Bicarbonate Haemodialysis and Long-Hour Slow-Flow Bicarbonate Haemodialysis. Nephrol. Dial. Transplant..

[45] Pawlak D., Tankiewicz A., Mysliwiec P., Buczko W. (2002). Tryptophan Metabolism via the Kynurenine Pathway in Experimental Chronic Renal Failure. Nephron.

[46] Opdebeeck B., Maudsley S., Azmi A., de Maré A., de Leger W., Meijers B., Verhulst A., Evenepoel P., D’Haese P.C., Neven E. (2019). Indoxyl Sulfate and P-Cresyl Sulfate Promote Vascular Calcification and Associate with Glucose Intolerance. J. Am. Soc. Nephrol..

[47] Barreto F.C., Barreto D.V., Liabeuf S., Meert N., Glorieux G., Temmar M., Choukroun G., Vanholder R., Massy Z.A. (2009). Serum Indoxyl Sulfate Is Associated with Vascular Disease and Mortality in Chronic Kidney Disease Patients. Clin. J. Am. Soc. Nephrol..

[48] Mozar A., Louvet L., Godin C., Mentaverri R., Brazier M., Kamel S., Massy Z.A. (2012). Indoxyl Sulphate Inhibits Osteoclast Differentiation and Function. Nephrol. Dial. Transplant..

[49] Shimizu H., Yisireyili M., Nishijima F., Niwa T. (2013). Indoxyl Sulfate Enhances p53-TGF-β1-Smad3 Pathway in Proximal Tubular Cells. Am. J. Nephrol..

[50] Dou L., Sallée M., Cerini C., Poitevin S., Gondouin B., Jourde-Chiche N., Fallague K., Brunet P., Calaf R., Dussol B. (2015). The Cardiovascular Effect of the Uremic Solute Indole-3 Acetic Acid. J. Am. Soc. Nephrol..

[51] Gondouin B., Cerini C., Dou L., Sallée M., Duval-Sabatier A., Pletinck A., Calaf R., Lacroix R., Jourde-Chiche N., Poitevin S. (2013). Indolic Uremic Solutes Increase Tissue Factor Production in Endothelial Cells by the Aryl Hydrocarbon Receptor Pathway. Kidney Int..

[52] Lin Y.-T., Wu P.-H., Lee H.-H., Mubanga M., Chen C.-S., Kuo M.-C., Chiu Y.-W., Kuo P.-L., Hwang S.-J. (2019). Indole-3 Acetic Acid Increased Risk of Impaired Cognitive Function in Patients Receiving Hemodialysis. Neurotoxicology.

[53] Pawlak K., Domaniewski T., Mysliwiec M., Pawlak D. (2009). The Kynurenines Are Associated with Oxidative Stress, Inflammation and the Prevalence of Cardiovascular Disease in Patients with End-Stage Renal Disease. Atherosclerosis.

[54] Karu N., McKercher C., Nichols D.S., Davies N., Shellie R.A., Hilder E.F., Jose M.D. (2016). Tryptophan Metabolism, Its Relation to Inflammation and Stress Markers and Association with Psychological and Cognitive Functioning: Tasmanian Chronic Kidney Disease Pilot Study. BMC Nephrol..

[55] Sun B., Wang X., Liu X., Wang L., Ren F., Wang X., Leng X. (2020). Hippuric Acid Promotes Renal Fibrosis by Disrupting Redox Homeostasis via Facilitation of NRF2–KEAP1–CUL3 Interactions in Chronic Kidney Disease. Antioxidants.

[56] Huang M., Wei R., Wang Y., Su T., Li P., Chen X. (2018). The Uremic Toxin Hippurate Promotes Endothelial Dysfunction via the Activation of Drp1-Mediated Mitochondrial Fission. Redox Biol..

[57] Glorieux G., Vanholder R., van Biesen W., Pletinck A., Schepers E., Neirynck N., Speeckaert M., de Bacquer D., Verbeke F. (2021). Free *p*-Cresyl Sulfate Shows the Highest Association with Cardiovascular Outcome in Chronic Kidney Disease. Nephrol. Dial. Transplant..

[58] Poveda J., Sanchez-Niño M.D., Glorieux G., Sanz A.B., Egido J., Vanholder R., Ortiz A. (2014). P-Cresyl Sulphate Has pro-Inflammatory and Cytotoxic Actions on Human Proximal Tubular Epithelial Cells. Nephrol. Dial. Transplant..

[59] Koppe L., Pillon N.J., Vella R.E., Croze M.L., Pelletier C.C., Chambert S., Massy Z., Glorieux G., Vanholder R., Dugenet Y. (2013). P-Cresyl Sulfate Promotes Insulin Resistance Associated with CKD. J. Am. Soc. Nephrol..

[60] Verbeke F., Vanholder R., van Biesen W., Glorieux G. (2022). Contribution of Hypoalbuminemia and Anemia to the Prognostic Value of Plasma P-Cresyl Sulfate and p-Cresyl Glucuronide for Cardiovascular Outcome in Chronic Kidney Disease. J. Pers. Med..

[61] Kim R.B., Morse B.L., Djurdjev O., Tang M., Muirhead N., Barrett B., Holmes D.T., Madore F., Clase C.M., Rigatto C. (2016). Advanced Chronic Kidney Disease Populations Have Elevated Trimethylamine N-Oxide Levels Associated with Increased Cardiovascular Events. Kidney Int..

[62] Gruppen E.G., Garcia E., Connelly M.A., Jeyarajah E.J., Otvos J.D., Bakker S.J.L., Dullaart R.P.F. (2017). TMAO Is Associated with Mortality: Impact of Modestly Impaired Renal Function. Sci. Rep..

[63] Fang Q., Zheng B., Liu N., Liu J., Liu W., Huang X., Zeng X., Chen L., Li Z., Ouyang D. (2021). Trimethylamine N-Oxide Exacerbates Renal Inflammation and Fibrosis in Rats With Diabetic Kidney Disease. Front. Physiol..

[64] Claro L., Moreno-Amaral A., Gadotti A., Dolenga C., Nakao L., Azevedo M., de Noronha L., Olandoski M., de Moraes T., Stinghen A. (2018). The Impact of Uremic Toxicity Induced Inflammatory Response on the Cardiovascular Burden in Chronic Kidney Disease. Toxins.

[65] Rossi M., Campbell K.L., Johnson D.W., Stanton T., Vesey D.A., Coombes J.S., Weston K.S., Hawley C.M., McWhinney B.C., Ungerer J.P.J. (2014). Protein-Bound Uremic Toxins, Inflammation and Oxidative Stress: A Cross-Sectional Study in Stage 3–4 Chronic Kidney Disease. Arch. Med. Res..

[66] Chen S.-C., Huang S.-Y., Wu C.-C., Hsu C.-F. (2020). P-Cresylsulfate, the Protein-Bound Uremic Toxin, Increased Endothelial Permeability Partly Mediated by Src-Induced Phosphorylation of VE-Cadherin. Toxins.

[67] Li S., Xie Y., Yang B., Huang S., Zhang Y., Jia Z., Ding G., Zhang A. (2020). MicroRNA-214 Targets COX-2 to Antagonize Indoxyl Sulfate (IS)-Induced Endothelial Cell Apoptosis. Apoptosis.

[68] Dou L., Jourde-Chiche N., Faure V., Cerini C., Berland Y., Dignat-George F., Brunet P. (2007). The Uremic Solute Indoxyl Sulfate Induces Oxidative Stress in Endothelial Cells. J. Thromb. Haemost..

[69] Yisireyili M., Shimizu H., Saito S., Enomoto A., Nishijima F., Niwa T. (2013). Indoxyl Sulfate Promotes Cardiac Fibrosis with Enhanced Oxidative Stress in Hypertensive Rats. Life Sci..

[70] Rodrigues G.G.C., Dellê H., Brito R.B.O., Cardoso V.O., Fernandes K.P.S., Mesquita-Ferrari R.A., Cunha R.S., Stinghen A.E.M., Dalboni M.A., Barreto F.C. (2020). Indoxyl Sulfate Contributes to Uremic Sarcopenia by Inducing Apoptosis in Myoblasts. Arch. Med. Res..

[71] Chen J., Zhang X., Zhang H., Liu T., Zhang H., Teng J., Ji J., Ding X. (2016). Indoxyl Sulfate Enhance the Hypermethylation of Klotho and Promote the Process of Vascular Calcification in Chronic Kidney Disease. Int. J. Biol. Sci..

[72] Yang K., Nie L., Huang Y., Zhang J., Xiao T., Guan X., Zhao J. (2012). Amelioration of Uremic Toxin Indoxyl Sulfate-Induced Endothelial Cell Dysfunction by Klotho Protein. Toxicol. Lett..

[73] Fourdinier O., Glorieux G., Brigant B., Diouf M., Pletinck A., Vanholder R., Choukroun G., Verbeke F., Massy Z.A., Metzinger-Le Meuth V. (2021). Syndecan-1 and Free Indoxyl Sulfate Levels Are Associated with MiR-126 in Chronic Kidney Disease. Int. J. Mol. Sci..

[74] Huang Y.-C., Tsai T.-C., Chang C.-H., Chang K.-T., Ko P.-H., Lai L.-C. (2021). Indoxyl Sulfate Elevated Lnc-SLC15A1-1 Upregulating CXCL10/CXCL8 Expression in High-Glucose Endothelial Cells by Sponging MicroRNAs. Toxins.

[75] Ito S., Osaka M., Higuchi Y., Nishijima F., Ishii H., Yoshida M. (2010). Indoxyl Sulfate Induces Leukocyte-Endothelial Interactions through Up-Regulation of E-Selectin. J. Biol. Chem..

[76] Li Y., Yan J., Wang M., Lv J., Yan F., Chen J. (2021). Uremic Toxin Indoxyl Sulfate Promotes Proinflammatory Macrophage Activation by Regulation of β-Catenin and YAP Pathways. J. Mol. Histol..

[77] Wang W.-J., Cheng M.-H., Sun M.-F., Hsu S.-F., Weng C.-S. (2014). Indoxyl Sulfate Induces Renin Release and Apoptosis of Kidney Mesangial Cells. J. Toxicol. Sci..

[78] Yang J., Li H., Zhang C., Zhou Y. (2022). Indoxyl Sulfate Reduces Ito,f by Activating ROS/MAPK and NF-ΚB Signaling Pathways. JCI Insight.

[79] Edamatsu T., Fujieda A., Itoh Y. (2018). Phenyl Sulfate, Indoxyl Sulfate and p-Cresyl Sulfate Decrease Glutathione Level to Render Cells Vulnerable to Oxidative Stress in Renal Tubular Cells. PLoS ONE.

[80] Watanabe H., Miyamoto Y., Honda D., Tanaka H., Wu Q., Endo M., Noguchi T., Kadowaki D., Ishima Y., Kotani S. (2013). P-Cresyl Sulfate Causes Renal Tubular Cell Damage by Inducing Oxidative Stress by Activation of NADPH Oxidase. Kidney Int..

[81] Bolati D., Shimizu H., Yisireyili M., Nishijima F., Niwa T. (2013). Indoxyl Sulfate, a Uremic Toxin, Downregulates Renal Expression of Nrf2 through Activation of NF-ΚB. BMC Nephrol..

[82] Salyers Z.R., Coleman M., Balestrieri N.P., Ryan T.E. (2021). Indoxyl Sulfate Impairs Angiogenesis via Chronic Aryl Hydrocarbon Receptor Activation. Am. J. Physiol. Cell Physiol..

[83] Miao J., Huang J., Luo C., Ye H., Ling X., Wu Q., Shen W., Zhou L. (2021). Klotho Retards Renal Fibrosis through Targeting Mitochondrial Dysfunction and Cellular Senescence in Renal Tubular Cells. Physiol. Rep..

[84] Sun C.-Y., Chang S.-C., Wu M.-S. (2012). Suppression of Klotho Expression by Protein-Bound Uremic Toxins Is Associated with Increased DNA Methyltransferase Expression and DNA Hypermethylation. Kidney Int..

[85] Uchiyama K., Wakino S., Irie J., Miyamoto J., Matsui A., Tajima T., Itoh T., Oshima Y., Yoshifuji A., Kimura I. (2020). Contribution of Uremic Dysbiosis to Insulin Resistance and Sarcopenia. Nephrol. Dial. Transplant..

[86] McCaleb M.L., Izzo M.S., Lockwood D.H. (1985). Characterization and Partial Purification of a Factor from Uremic Human Serum That Induces Insulin Resistance. J. Clin. Investig..

[87] Stockler-Pinto M.B., Saldanha J.F., Yi D., Mafra D., Fouque D., Soulage C.O. (2016). The Uremic Toxin Indoxyl Sulfate Exacerbates Reactive Oxygen Species Production and Inflammation in 3T3-L1 Adipose Cells. Free Radic. Res..

[88] Afsar B., Elsurer R., Covic A., Johnson R.J., Kanbay M. (2011). Relationship between Uric Acid and Subtle Cognitive Dysfunction in Chronic Kidney Disease. Am. J. Nephrol..

[89] Vannorsdall T.D., Jinnah H.A., Gordon B., Kraut M., Schretlen D.J. (2008). Cerebral Ischemia Mediates the Effect of Serum Uric Acid on Cognitive Function. Stroke.

[90] Sharma M., Zhou Z., Miura H., Papapetropoulos A., McCarthy E.T., Sharma R., Savin V.J., Lianos E.A. (2009). ADMA Injures the Glomerular Filtration Barrier: Role of Nitric Oxide and Superoxide. Am. J. Physiol. Ren. Physiol..

[91] Ohtsuki S., Asaba H., Takanaga H., Deguchi T., Hosoya K., Otagiri M., Terasaki T. (2002). Role of Blood-Brain Barrier Organic Anion Transporter 3 (OAT3) in the Efflux of Indoxyl Sulfate, a Uremic Toxin: Its Involvement in Neurotransmitter Metabolite Clearance from the Brain. J. Neurochem..

[92] Watanabe K., Sato E., Mishima E., Watanabe M., Abe T., Takahashi N., Nakayama M. (2021). Effect of Uremic Toxins on Hippocampal Cell Damage: Analysis in Vitro and in Rat Model of Chronic Kidney Disease. Heliyon.

[93] Chen C.-H., Huang S.-C., Yeh E.-L., Lin P.-C., Tsai S.-F., Huang Y.-C. (2022). Indoxyl Sulfate, Homocysteine, and Antioxidant Capacities in Patients at Different Stages of Chronic Kidney Disease. Nutr. Res. Pract..

[94] Rydzewska-Rosołowska A., Sroka N., Kakareko K., Rosołowski M., Zbroch E., Hryszko T. (2020). The Links between Microbiome and Uremic Toxins in Acute Kidney Injury: Beyond Gut Feeling—A Systematic Review. Toxins.

[95] Kurella Tamura M., Tam K., Vittinghoff E., Raj D., Sozio S.M., Rosas S.E., Makos G., Lora C., He J., Go A.S. (2017). Inflammatory Markers and Risk for Cognitive Decline in Chronic Kidney Disease: The CRIC Study. Kidney Int. Rep..

[96] Fukui S., Schwarcz R., Rapoport S.I., Takada Y., Smith Q.R. (1991). Blood?Brain Barrier Transport of Kynurenines: Implications for Brain Synthesis and Metabolism. J. Neurochem..

[97] Okuda S., Nishiyama N., Saito H., Katsuki H. (2002). 3-Hydroxykynurenine, an Endogenous Oxidative Stress Generator, Causes Neuronal Cell Death with Apoptotic Features and Region Selectivity. J. Neurochem..

[98] Reyes-Ocampo J., Ramírez-Ortega D., Vázquez Cervantes G.I., Pineda B., Montes de Oca Balderas P., González-Esquivel D., Sánchez-Chapul L., Lugo-Huitrón R., Silva-Adaya D., Ríos C. (2015). Mitochondrial Dysfunction Related to Cell Damage Induced by 3-Hydroxykynurenine and 3-Hydroxyanthranilic Acid: Non-Dependent-Effect of Early Reactive Oxygen Species Production. Neurotoxicology.

[99] Guillemin G.J., Smith D.G., Smythe G.A., Armati P.J., Brew G.J. (2003). Expression of The Kynurenine Pathway Enzymes in Human Microglia and Macrophages. Developments in Tryptophan and Serotonin Metabolism.

[100] Lee M.-C., Ting K.K., Adams S., Brew B.J., Chung R., Guillemin G.J. (2010). Characterisation of the Expression of NMDA Receptors in Human Astrocytes. PLoS ONE.

[101] Guillemin G.J., Wang L., Brew B.J. (2005). Quinolinic Acid Selectively Induces Apoptosis of Human Astrocytes: Potential Role in AIDS Dementia Complex. J. Neuroinflamm..

[102] Jang H.R., Gandolfo M.T., Ko G.J., Satpute S., Racusen L., Rabb H. (2009). Early Exposure to Germs Modifies Kidney Damage and Inflammation after Experimental Ischemia-Reperfusion Injury. Am. J. Physiol. Ren. Physiol..

[103] Samanta A., Patra A., Mandal S., Roy S., Das K., Kar S., Nandi D. (2018). Hypoxia: A Cause of Acute Renal Failure and Alteration of Gastrointestinal Microbial Ecology. Saudi J. Kidney Dis. Transplant..

[104] Kalim S., Clish C.B., Deferio J.J., Ortiz G., Moffet A.S., Gerszten R.E., Thadhani R., Rhee E.P. (2015). Cross-Sectional Examination of Metabolites and Metabolic Phenotypes in Uremia. BMC Nephrol..

[105] Wang W., Hao G., Pan Y., Ma S., Yang T., Shi P., Zhu Q., Xie Y., Ma S., Zhang Q. (2019). Serum Indoxyl Sulfate Is Associated with Mortality in Hospital-Acquired Acute Kidney Injury: A Prospective Cohort Study. BMC Nephrol..

[106] Veldeman L., Vanmassenhove J., van Biesen W., Massy Z.A., Liabeuf S., Glorieux G., Vanholder R. (2019). Evolution of Protein-Bound Uremic Toxins Indoxyl Sulphate and p-Cresyl Sulphate in Acute Kidney Injury. Int. Urol. Nephrol..

[107] Devlin A.S., Marcobal A., Dodd D., Nayfach S., Plummer N., Meyer T., Pollard K.S., Sonnenburg J.L., Fischbach M.A. (2016). Modulation of a Circulating Uremic Solute via Rational Genetic Manipulation of the Gut Microbiota. Cell Host Microbe.

[108] Zhou X., Yao J., Lin J., Liu J., Dong L., Duan M. (2022). Th17/Regulatory T-Cell Imbalance and Acute Kidney Injury in Patients with Sepsis. J. Clin. Med..

[109] Dong T., Aronsohn A., Gautham Reddy K., Te H.S. (2016). Rifaximin Decreases the Incidence and Severity of Acute Kidney Injury and Hepatorenal Syndrome in Cirrhosis. Dig. Dis. Sci..

[110] Lee J.R., Magruder M., Zhang L., Westblade L.F., Satlin M.J., Robertson A., Edusei E., Crawford C., Ling L., Taur Y. (2019). Gut Microbiota Dysbiosis and Diarrhea in Kidney Transplant Recipients. Am. J. Transplant..

[111] Winichakoon P., Chaiwarith R., Chattipakorn N., Chattipakorn S.C. (2022). Impact of Gut Microbiota on Kidney Transplantation. Transplant. Rev..

[112] Ahmad S., Bromberg J.S. (2016). Current Status of the Microbiome in Renal Transplantation. Curr. Opin. Nephrol. Hypertens..

[113] Pletinck A., Glorieux G., Schepers E., Cohen G., Gondouin B., van Landschoot M., Eloot S., Rops A., van de Voorde J., de Vriese A. (2013). Protein-Bound Uremic Toxins Stimulate Crosstalk between Leukocytes and Vessel Wall. J. Am. Soc. Nephrol..

[114] Liabeuf S., Barreto D.V., Barreto F.C., Meert N., Glorieux G., Schepers E., Temmar M., Choukroun G., Vanholder R., Massy Z.A. (2010). Free P-Cresylsulphate Is a Predictor of Mortality in Patients at Different Stages of Chronic Kidney Disease. Nephrol. Dial. Transplant..

[115] Lin C.-J., Wu V., Wu P.-C., Wu C.-J. (2015). Meta-Analysis of the Associations of p-Cresyl Sulfate (PCS) and Indoxyl Sulfate (IS) with Cardiovascular Events and All-Cause Mortality in Patients with Chronic Renal Failure. PLoS ONE.

[116] Liabeuf S., Desjardins L., Massy Z.A., Brazier F., Westeel P.F., Mazouz H., Titeca-Beauport D., Diouf M., Glorieux G., Vanholder R. (2016). Levels of Indoxyl Sulfate in Kidney Transplant Patients, and the Relationship With Hard Outcomes. Circ. J..

[117] Poesen R., Evenepoel P., de Loor H., Bammens B., Claes K., Sprangers B., Naesens M., Kuypers D., Augustijns P., Meijers B. (2016). The Influence of Renal Transplantation on Retained Microbial–Human Co-Metabolites. Nephrol. Dial. Transplant..

[118] Te Linde E., van Roij C.J.M., Meijers B.K.I., de Loor H., Kessels R.P.C., Wetzels J.F.M. (2020). Cognitive Function and Uremic Toxins after Kidney Transplantation: An Exploratory Study. Kidney360.

[119] Yu Y., Guan X., Nie L., Liu Y., He T., Xiong J., Xu X., Li Y., Yang K., Wang Y. (2017). DNA Hypermethylation of SFRP5 Contributes to Indoxyl Sulfate-Induced Renal Fibrosis. J. Mol. Med..

[120] Korytowska N., Wyczałkowska-Tomasik A., Pączek L., Giebułtowicz J. (2021). Evaluation of Salivary Indoxyl Sulfate with Proteinuria for Predicting Graft Deterioration in Kidney Transplant Recipients. Toxins.

[121] Wolf M., Molnar M.Z., Amaral A.P., Czira M.E., Rudas A., Ujszaszi A., Kiss I., Rosivall L., Kosa J., Lakatos P. (2011). Elevated Fibroblast Growth Factor 23 Is a Risk Factor for Kidney Transplant Loss and Mortality. J. Am. Soc. Nephrol..

[122] Frenay A.-R.S., van den Berg E., de Borst M.H., Beckmann B., Tsikas D., Feelisch M., Navis G., Bakker S.J.L., van Goor H. (2015). Plasma ADMA Associates with All-Cause Mortality in Renal Transplant Recipients. Amino Acids.

[123] Eiseman B., Silen W., Bascom G.S., Kauvar A.J. (1958). Fecal Enema as an Adjunct in the Treatment of Pseudomembranous Enterocolitis. Surgery.

[124] Wang J.-W., Kuo C.-H., Kuo F.-C., Wang Y.-K., Hsu W.-H., Yu F.-J., Hu H.-M., Hsu P.-I., Wang J.-Y., Wu D.-C. (2019). Fecal Microbiota Transplantation: Review and Update. J. Formos. Med. Assoc..

[125] Zipursky J.S., Sidorsky T.I., Freedman C.A., Sidorsky M.N., Kirkland K.B. (2012). Patient Attitudes Toward the Use of Fecal Microbiota Transplantation in the Treatment of Recurrent *Clostridium difficile* Infection. Clin. Infect. Dis..

[126] Cammarota G., Ianiro G., Tilg H., Rajilić-Stojanović M., Kump P., Satokari R., Sokol H., Arkkila P., Pintus C., Hart A. (2017). European Consensus Conference on Faecal Microbiota Transplantation in Clinical Practice. Gut.

[127] Bhutiani N., Schucht J.E., Miller K.R., McClave S.A. (2018). Technical Aspects of Fecal Microbial Transplantation (FMT). Curr. Gastroenterol. Rep..

[128] Kao D., Roach B., Silva M., Beck P., Rioux K., Kaplan G.G., Chang H.-J., Coward S., Goodman K.J., Xu H. (2017). Effect of Oral Capsule- vs Colonoscopy-Delivered Fecal Microbiota Transplantation on Recurrent *Clostridium difficile* Infection. JAMA.

[129] Hirsch B.E., Saraiya N., Poeth K., Schwartz R.M., Epstein M.E., Honig G. (2015). Effectiveness of Fecal-Derived Microbiota Transfer Using Orally Administered Capsules for Recurrent *Clostridium difficile* Infection. BMC Infect. Dis..

[130] Varga A., Kocsis B., Sipos D., Kása P., Vigvári S., Pál S., Dembrovszky F., Farkas K., Péterfi Z. (2021). How to Apply FMT More Effectively, Conveniently and Flexible—A Comparison of FMT Methods. Front. Cell. Infect. Microbiol..

[131] Du C., Luo Y., Walsh S., Grinspan A. (2021). Oral Fecal Microbiota Transplant Capsules Are Safe and Effective for Recurrent Clostridioides Difficile Infection. J. Clin. Gastroenterol..

[132] Chehri M., Christensen A.H., Halkjær S.I., Günther S., Petersen A.M., Helms M. (2018). Case Series of Successful Treatment with Fecal Microbiota Transplant (FMT) Oral Capsules Mixed from Multiple Donors Even in Patients Previously Treated with FMT Enemas for Recurrent *Clostridium difficile* Infection. Medicine.

[133] Youngster I., Russell G.H., Pindar C., Ziv-Baran T., Sauk J., Hohmann E.L. (2014). Oral, Capsulized, Frozen Fecal Microbiota Transplantation for Relapsing *Clostridium difficile* Infection. JAMA.

[134] Takkavatakarn K., Wuttiputinun T., Phannajit J., Praditpornsilpa K., Eiam-Ong S., Susantitaphong P. (2021). Protein-Bound Uremic Toxin Lowering Strategies in Chronic Kidney Disease: A Systematic Review and Meta-Analysis. J. Nephrol..

[135] Liu X., Zhang M., Wang X., Liu P., Wang L., Li Y., Wang X., Ren F. (2022). Fecal Microbiota Transplantation Restores Normal Fecal Composition and Delays Malignant Development of Mild Chronic Kidney Disease in Rats. Front. Microbiol..

[136] Barba C., Soulage C.O., Caggiano G., Glorieux G., Fouque D., Koppe L. (2020). Effects of Fecal Microbiota Transplantation on Composition in Mice with CKD. Toxins.

[137] Hu Z.B., Lu J., Chen P.P., Lu C.C., Zhang J.X., Li X.Q., Yuan B.Y., Huang S.J., Ruan X.Z., Liu B.C. (2020). Dysbiosis of Intestinal Microbiota Mediates Tubulointerstitial Injury in Diabetic Nephropathy via the Disruption of Cholesterol Homeostasis. Theranostics.

[138] Lu J., Chen P.P., Zhang J.X., Li X.Q., Wang G.H., Yuan B.Y., Huang S.J., Liu X.Q., Jiang T.T., Wang M.Y. (2021). GPR43 Deficiency Protects against Podocyte Insulin Resistance in Diabetic Nephropathy through the Restoration of AMPKα Activity. Theranostics.

[139] Bastos R.M.C., Simplício-Filho A., Sávio-Silva C., Oliveira L.F.V., Cruz G.N.F., Sousa E.H., Noronha I.L., Mangueira C.L.P., Quaglierini-Ribeiro H., Josefi-Rocha G.R. (2022). Fecal Microbiota Transplant in a Pre-Clinical Model of Type 2 Diabetes Mellitus, Obesity and Diabetic Kidney Disease. Int. J. Mol. Sci..

[140] Lauriero G., Abbad L., Vacca M., Celano G., Chemouny J.M., Calasso M., Berthelot L., Gesualdo L., de Angelis M., Monteiro R.C. (2021). Fecal Microbiota Transplantation Modulates Renal Phenotype in the Humanized Mouse Model of IgA Nephropathy. Front. Immunol..

[141] Emal D., Rampanelli E., Stroo I., Butter L.M., Teske G.J., Claessen N., Stokman G., Florquin S., Leemans J.C., Dessing M.C. (2017). Depletion of Gut Microbiota Protects against Renal Ischemia-Reperfusion Injury. J. Am. Soc. Nephrol..

[142] Nakade Y., Iwata Y., Furuichi K., Mita M., Hamase K., Konno R., Miyake T., Sakai N., Kitajima S., Toyama T. (2018). Gut Microbiota–Derived D-Serine Protects against Acute Kidney Injury. JCI Insight.

[143] Zhao J., Bai M., Yang X., Wang Y., Li R., Sun S. (2021). Alleviation of Refractory IgA Nephropathy by Intensive Fecal Microbiota Transplantation: The First Case Reports. Ren. Fail..

[144] Zhou G., Zeng J., Peng L., Wang L., Zheng W., Wu D., Yang Y. (2021). Fecal Microbiota Transplantation for Membranous Nephropathy. CEN Case Rep..

[145] Zhi W., Yuan X., Song W., Jin G., Li Y. (2022). Fecal Microbiota Transplantation May Represent a Good Approach for Patients with Focal Segmental Glomerulosclerosis: A Brief Report. J. Clin. Med..

[146] Cai T.-T., Ye X.-L., Li R.-R., Chen H., Wang Y.-Y., Yong H.-J., Pan M.-L., Lu W., Tang Y., Miao H. (2020). Resveratrol Modulates the Gut Microbiota and Inflammation to Protect Against Diabetic Nephropathy in Mice. Front. Pharmacol..

[147] Han C., Jiang Y., Li W., Liu Y. (2021). Astragalus Membranaceus and Salvia Miltiorrhiza Ameliorates Cyclosporin A-Induced Chronic Nephrotoxicity through the “Gut-Kidney Axis”. J. Ethnopharmacol..

[148] Zheng D.-W., Pan P., Chen K.-W., Fan J.-X., Li C.-X., Cheng H., Zhang X.-Z. (2020). An Orally Delivered Microbial Cocktail for the Removal of Nitrogenous Metabolic Waste in Animal Models of Kidney Failure. Nat. Biomed. Eng..

[149] Yu D.H., Ying N., Lian Z.H., Fa Y.Q. (2021). The Alteration Human of Gut Microbiota and Metabolites before and after Renal Transplantation. Microb. Pathog..

[150] Guirong Y.E., Minjie Z., Lixin Y.U., Junsheng Y.E., Lin Y., Lisha S. (2018). [Gut Microbiota in Renal Transplant Recipients, Patients with Chronic Kidney Disease and Healthy Subjects]. Nan Fang Yi Ke Da Xue Xue Bao.

[151] Zaza G., Dalla Gassa A., Felis G., Granata S., Torriani S., Lupo A. (2017). Impact of Maintenance Immunosuppressive Therapy on the Fecal Microbiome of Renal Transplant Recipients: Comparison between an Everolimus- and a Standard Tacrolimus-Based Regimen. PLoS ONE.

[152] Wu H., Noordmans G.A., O’Brien M.R., Ma J., Zhao C.Y., Zhang G.Y., Kwan T.K.T., Alexander S.I., Chadban S.J. (2012). Absence of MyD88 Signaling Induces Donor-Specific Kidney Allograft Tolerance. J. Am. Soc. Nephrol..

[153] Magruder M., Edusei E., Zhang L., Albakry S., Satlin M.J., Westblade L.F., Malha L., Sze C., Lubetzky M., Dadhania D.M. (2020). Gut Commensal Microbiota and Decreased Risk for *Enterobacteriaceae* Bacteriuria and Urinary Tract Infection. Gut Microbes.

[154] Carron C., Pais de Barros J.-P., Gaiffe E., Deckert V., Adda-Rezig H., Roubiou C., Laheurte C., Masson D., Simula-Faivre D., Louvat P. (2019). End-Stage Renal Disease-Associated Gut Bacterial Translocation: Evolution and Impact on Chronic Inflammation and Acute Rejection After Renal Transplantation. Front. Immunol..

[155] Stripling J., Kumar R., Baddley J.W., Nellore A., Dixon P., Howard D., Ptacek T., Lefkowitz E.J., Tallaj J.A., Benjamin W.H. (2015). Loss of Vancomycin-Resistant Enterococcus Fecal Dominance in an Organ Transplant Patient With *Clostridium difficile* Colitis After Fecal Microbiota Transplant. Open Forum Infect. Dis..

[156] Tariq R., Pardi D.S., Tosh P.K., Walker R.C., Razonable R.R., Khanna S. (2017). Fecal Microbiota Transplantation for Recurrent *Clostridium difficile* Infection Reduces Recurrent Urinary Tract Infection Frequency. Clin. Infect. Dis..

[157] Koppe L., Croze M.L., Monteiro E.B., Benoit B., Bres E., Guebre-Egziabher F., Daleprane J.B., Fouque D., Soulage C.O. (2021). The Protein-Bound Uremic Toxin p-Cresyl-Sulfate Promotes Intracellular ROS Production and Lipid Peroxidation in 3T3-L1 Adipose Cells. Biochimie.

[158] Prochazkova P., Roubalova R., Dvorak J., Tlaskalova-Hogenova H., Cermakova M., Tomasova P., Sediva B., Kuzma M., Bulant J., Bilej M. (2019). Microbiota, Microbial Metabolites, and Barrier Function in A Patient with Anorexia Nervosa after Fecal Microbiota Transplantation. Microorganisms.

[159] Pérez-Matute P., Íñiguez M., de Toro M., Recio-Fernández E., Oteo J.A. (2020). Autologous Fecal Transplantation from a Lean State Potentiates Caloric Restriction Effects on Body Weight and Adiposity in Obese Mice. Sci. Rep..

[160] Hu X.-F., Zhang W.-Y., Wen Q., Chen W.-J., Wang Z.-M., Chen J., Zhu F., Liu K., Cheng L.-X., Yang J. (2019). Fecal Microbiota Transplantation Alleviates Myocardial Damage in Myocarditis by Restoring the Microbiota Composition. Pharmacol. Res..

[161] Burrello C., Garavaglia F., Cribiù F.M., Ercoli G., Lopez G., Troisi J., Colucci A., Guglietta S., Carloni S., Guglielmetti S. (2018). Therapeutic Faecal Microbiota Transplantation Controls Intestinal Inflammation through IL10 Secretion by Immune Cells. Nat. Commun..

[162] Wang Y., Ren R., Sun G., Peng L., Tian Y., Yang Y. (2020). Pilot Study of Cytokine Changes Evaluation after Fecal Microbiota Transplantation in Patients with Ulcerative Colitis. Int. Immunopharmacol..

[163] Li S., Lv J., Li J., Zhao Z., Guo H., Zhang Y., Cheng S., Sun J., Pan H., Fan S. (2018). Intestinal Microbiota Impact Sepsis Associated Encephalopathy via the Vagus Nerve. Neurosci. Lett..

[164] Karbach S.H., Schönfelder T., Brandão I., Wilms E., Hörmann N., Jäckel S., Schüler R., Finger S., Knorr M., Lagrange J. (2016). Gut Microbiota Promote Angiotensin II–Induced Arterial Hypertension and Vascular Dysfunction. J. Am. Heart Assoc..

[165] Jiang S., Shui Y., Cui Y., Tang C., Wang X., Qiu X., Hu W., Fei L., Li Y., Zhang S. (2021). Gut Microbiota Dependent Trimethylamine N-Oxide Aggravates Angiotensin II–Induced Hypertension. Redox Biol..

[166] Sun C.-Y., Chang S.-C., Wu M.-S. (2012). Uremic Toxins Induce Kidney Fibrosis by Activating Intrarenal Renin–Angiotensin–Aldosterone System Associated Epithelial-to-Mesenchymal Transition. PLoS ONE.

